# A critical period for faces: Other-race face recognition is improved by childhood but not adult social contact

**DOI:** 10.1038/s41598-019-49202-0

**Published:** 2019-09-06

**Authors:** Elinor McKone, Lulu Wan, Madeleine Pidcock, Kate Crookes, Katherine Reynolds, Amy Dawel, Evan Kidd, Chiara Fiorentini

**Affiliations:** 1 Research School of Psychology, Australian National University, Canberra, Australia; 2 ARC Centre of Excellence for Cognition and Its Disorders, Australian National University, Canberra, Australia; 3 School of Psychological Science and ARC Centre of Excellent for Cognition and Its Disorders, University of Western Australia, Perth, Australia; 4 ARC Centre of Excellence for the Dynamics of Language, Australian National University, Canberra, Australia; 5 Max Planck Institute for Psycholinguistics, Nijmegen, Netherlands

**Keywords:** Perception, Human behaviour

## Abstract

Poor recognition of other-race faces is ubiquitous around the world. We resolve a longstanding contradiction in the literature concerning whether interracial social contact improves the other-race effect. For the first time, we measure the *age* at which contact was experienced. Taking advantage of unusual demographics allowing dissociation of childhood from adult contact, results show sufficient childhood contact eliminated poor other-race recognition altogether (confirming inter-country adoption studies). Critically, however, *the developmental window for easy acquisition of other-race faces closed by approximately 12 years of age* and social contact as an adult — even over several years and involving many other-race friends — produced *no* improvement. Theoretically, this pattern of developmental change in plasticity mirrors that found in language, suggesting a shared origin grounded in the functional importance of both skills to social communication. Practically, results imply that, where parents wish to ensure their offspring develop the perceptual skills needed to recognise other-race people easily, childhood experience should be encouraged: just as an English-speaking person who moves to France as a child (but not an adult) can easily become a native speaker of French, we can easily become “native recognisers” of other-race faces via natural social exposure obtained in childhood, but not later.

## Introduction

Poorer recognition of other-race faces than own-race faces^1^ (the *other-race effect*, ORE) is a problem of substantial real-world impact, contributing to difficulties in social interaction, inaccurate eyewitness testimony, implicit racism, and inaccurate face-to-photo matching in security settings^2–4^. How, then, can we best ensure that people are able to successfully recognise individuals of a different race or ethnicity to themselves?

Longstanding debate has focussed on a distinction between perceptually-based theories of ORE origin, which imply *additional experience* is the key factor (e.g., because ‘face-space’ dimensions are tuned by experience primarily with own-race faces, providing poor coding of other-race faces^5^), and theories based in social outgrouping, which imply that *improving attitudes or effort* are key (e.g., due to a hypothesised lack of motivation for other-race faces, or attention to race-category rather than individual-identity level processing^6^). Recent attempts at reconciling these two extremes have proposed that increasing motivation may be helpful only as long as a sufficient minimum degree of perceptual experience is present^6^, or that motivation contributes over-and-above experience only in cultural settings where the groups differ in socioeconomic status^7^.

Most of these theories predict that perceptual experience is important. Yet, surprisingly, the core prediction of the perceptual-experience idea — that the size of the ORE will vary with the amount of interracial contact experienced in everyday life — lacks convincing empirical support. Indeed, a longstanding contradiction is present in the many previous studies examining the correlation of the ORE with self-reported real-world contact. Some studies find clear correlations of the ORE with such contact^7–9^. However, others do not^10–13^, and one meta-analysis concluded that contact explained only 2% of variation in the size of the ORE^1^.

Here, we propose these contradictory findings may be due to an unmeasured variable, namely the *age* at which the contact occurred. Specifically, we hypothesise that *childhood contact may be more important than adult contact* for avoiding the ORE.

**Figure 1:**
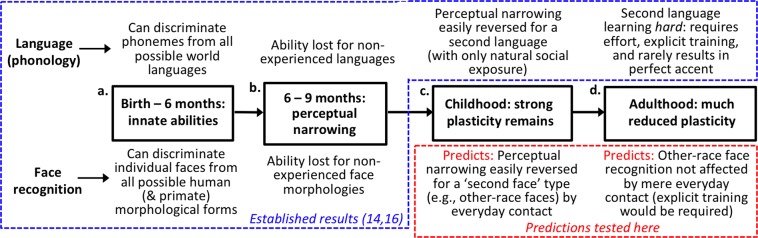
Parallel courses of developmental plasticity for faces and language.

We derive this hypothesis by proposing that developmental changes in plasticity for faces might mirror those established for language, consistent with a shared evolutionary role of faces and speech in social communication. As illustrated in Fig. 1, strong parallels between faces and aspects of language exist during infancy^14,15^. We test, for the first time, whether these parallels continue into subsequent developmental stages.

For school-aged children, at primary (elementary) school age, English speakers who move to France pick up French easily, with excellent accent and grammar, simply through ordinary social exposure. In contrast, second language learning is more difficult if exposure does not begin until adulthood: it typically requires explicit teaching, and even decades of real-world exposure typically does not produce a native-speaker accent. This is consistent with a critical period of enhanced plasticity in childhood^16,17^.

We ask whether face recognition might follow a similar developmental course, with other-race faces playing the role of the second, previously non-experienced, language (Fig. 1, red dotted region). Cross-continent adoption studies have already established that the other-race effect is sensitive to post-infancy childhood exposure: for example, Koreans adopted to France aged 3–9 years show no or even a reversed ORE^18,19^. However, no studies have assessed whether there might be a *critical period* for this contact, in which social experience is more effective when obtained before a certain age in development.

**Figure 2:**
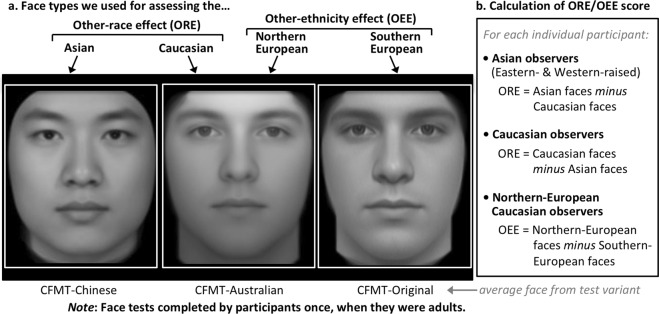
Races (continent of ancestry) and within-race ethnicities of face stimuli and observers. (**a**) Average face for three variants of the Cambridge Face Memory Test that display: Asian faces (CFMT-Chinese^24^); Caucasian faces of largely Northern-European appearance (CFMT-Australian^25^); and Caucasian faces of more Southern-European appearance (CFMT-original^20^). Note the multiple physical differences in both local features and aspects of global face structure; to facilitate comparison, white boxes are identical and images matched for distance between eyes. (**b**) Difference-score formulae used to calculate each participant’s other-race effect (ORE) or other-ethnicity effect (OEE) score.

**Figure 3:**
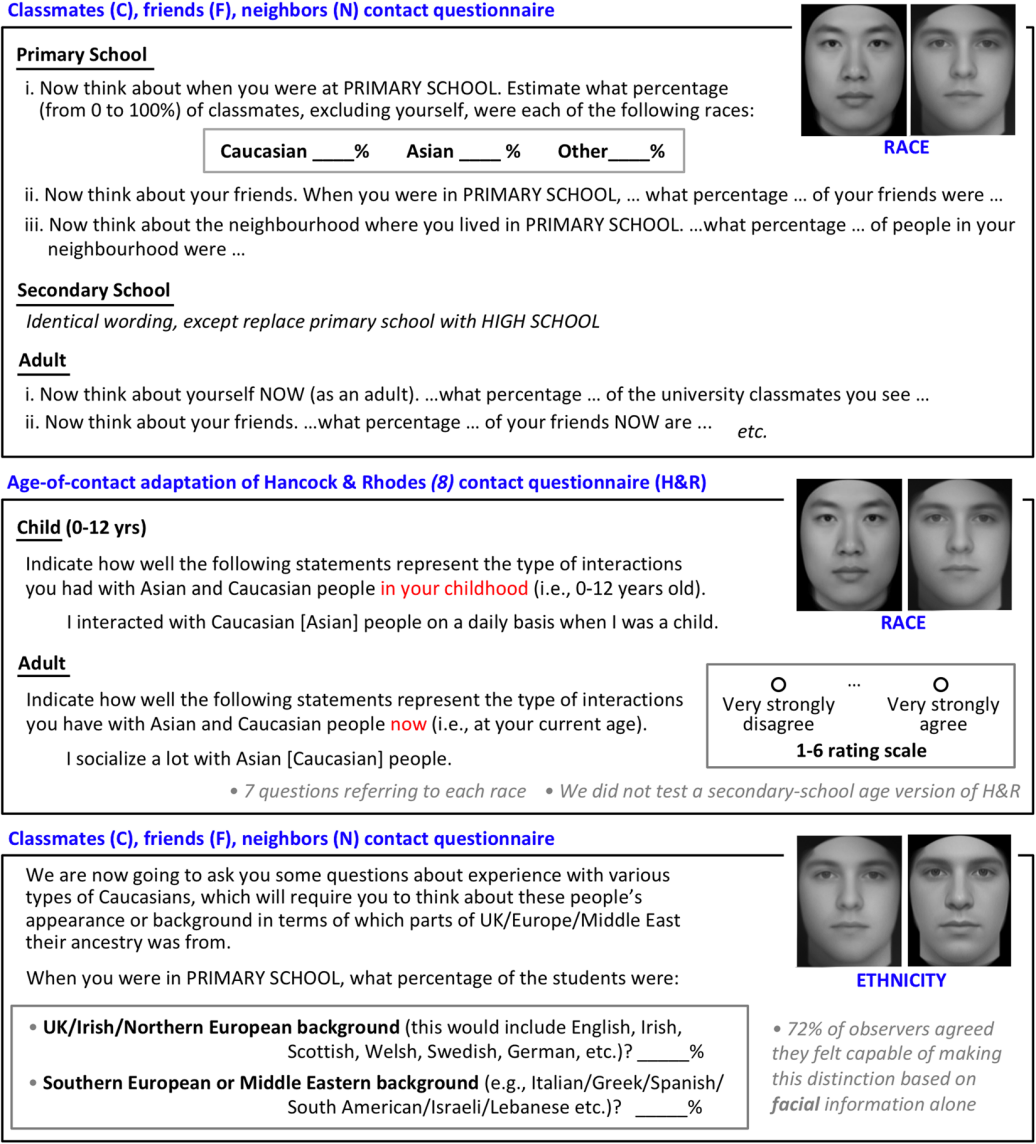
Our contact measures. We also measured time since moving to the West (Time-in-West) for Eastern-raised Asian participants. Intercorrelations between contact measures (Tables S15–17) were generally modest, as expected given that different measures tap different theoretical constructs (e.g., high quality contact vs mere exposure). Also note own- and other- contact are not merely opposites (see Tables S15–S17; this is because social contact can also include third-party groups (e.g., Indian, Indigenous Australian, African).

Here, we are the first to examine the correlation of people’s adult ORE with their self-reported contact based on the *age* at which the social contact was experienced. We measure the ORE, in adult participants, using own- and other-group variants of the Cambridge Face Memory Test^20^ (Fig. 2). We then examine the correlation of people’s adult ORE with the amount of natural social contact with own-race people, and the target group of other-race people, they report having previously experienced *at different ages* (Fig. 3). We also validate our contact measures by including a measure of prejudice (willingness to marry an other-race person), to ensure we can replicate the standard finding that higher other-race contact correlates with reduced prejudice^21–23^.

Our key prediction is that *age*-of-contact should matter to own-versus-other face recognition ability. Specifically, if there is greater plasticity of face recognition in childhood than in adulthood, then we predict a person’s adult ORE should correlate more strongly with their childhood social contact than their adult social contact. We test replicability of results concerning this prediction across 4 independent samples of participants, covering multiple situations. These include: people raised in own-race-majority countries; people raised in other-race-majority countries; two different races of observers (Asian, Caucasian); and two degrees of morphological difference between observers and faces, differing in *race* defined as continent of ancestry (Asia, Europe), and differing in *ethnicity* defined as smaller within-continent differences in ancestry (Northern vs Southern Europe; Fig. 2).

## Results

Face recognition performance was assessed when participants were adults, using race- and ethnicity-specific variants^24,25^ of the Cambridge Face Memory Test (CFMT^20^; Fig. 2a). For each observer, a difference score measure of their other-race effect or other-ethnicity effect was calculated (Fig. 2b); using a difference score for the ORE^7–13^ removes variance in raw CFMT scores that is due to general cognitive factors (e.g., general visual memory ability), giving a purer measure of race-specific face ability and improving statistical power. We divided contact measures (Fig. 3) into three age ranges chosen to refer to life stages that our young adult participants ought to be able to clearly distinguish in memory, specifically: contact obtained between 5 and 12 years of age (primary school), between 12–18 years (secondary school), and recently as adults (post-school). Because some researchers have theorised that “high-quality” contact might be more effective in reducing the ORE than mere exposure^5^ (although see contradictory evidence in^8,26,27^), we measured: contact with *friends* (high-quality contact requiring individuation and including positive social interactions); contact with *classmates* (requiring individuation but not necessarily positive social interactions); and exposure within the general *neighbourhood* (assessing incidental exposure).

Importantly, separating the effects of experience obtained at different ages requires that contact cannot be simply stable across the lifespan: instead a sample needs to have sufficient dissociation (i.e., a fairly low correlation) between contact in childhood and contact in adulthood. Testing in Australia, we were able to access four demographic samples (Fig. 2b; total *N* = 373) that met this criterion (Table 1; Methods). For the *other-race* effect, samples were: *Eastern-raised Asians* who moved to the West as adults; *Western-raised Asians* who were born and raised in Australia; and *Caucasians* who were Western-raised. These samples cover two races of observers (Asians, Caucasians), and groups raised in both own-race-majority and other-race-majority countries. For the *other-ethnicity* effect (OEE), our sample were *Northern-European Caucasians* of UK heritage.

**Table 1:** Correlation between primary school and adult contact, showing that, as required to evaluate the effect of contact at different life stages, contact is not simply stable across the lifespan (i.e., correlations are low).

Observer Group	Contact measure	Correlation of Primary with Adult contact	tau/r
Caucasians for other-race contact	Class	−0.04	r
	Friends	0.22	tau
	Neighbours	0.03	tau
	H&R	0.25	r
Western-raised Asians for other-race contact	Class	0.26	tau
	Friends	0.44	r
	Neighbours	0.41	tau
	H&R	0.19	tau
Northern-Europeans for other-ethnicity contact	Class	0.21	tau
	Friends	0.38	tau

Note: Observer group Eastern-raised Asians was not analysable formally because their primary school other-race contact was very close to zero, but this in itself indicates the desired independence between childhood and adult contact, given the wide range of adult contact scores in this group (Table S4). Choice of *tau* vs *r* to evaluate correlation determined by level of skew in contact distributions (Tables S2 and S4).

Within each sample, we calculated correlations between contact (with both own-group and target other-group people) reported at different stages of development, and the observer’s eventual adult ORE or OEE.

### Is there a critical period? Contact at different life stages

**Figure 4:**
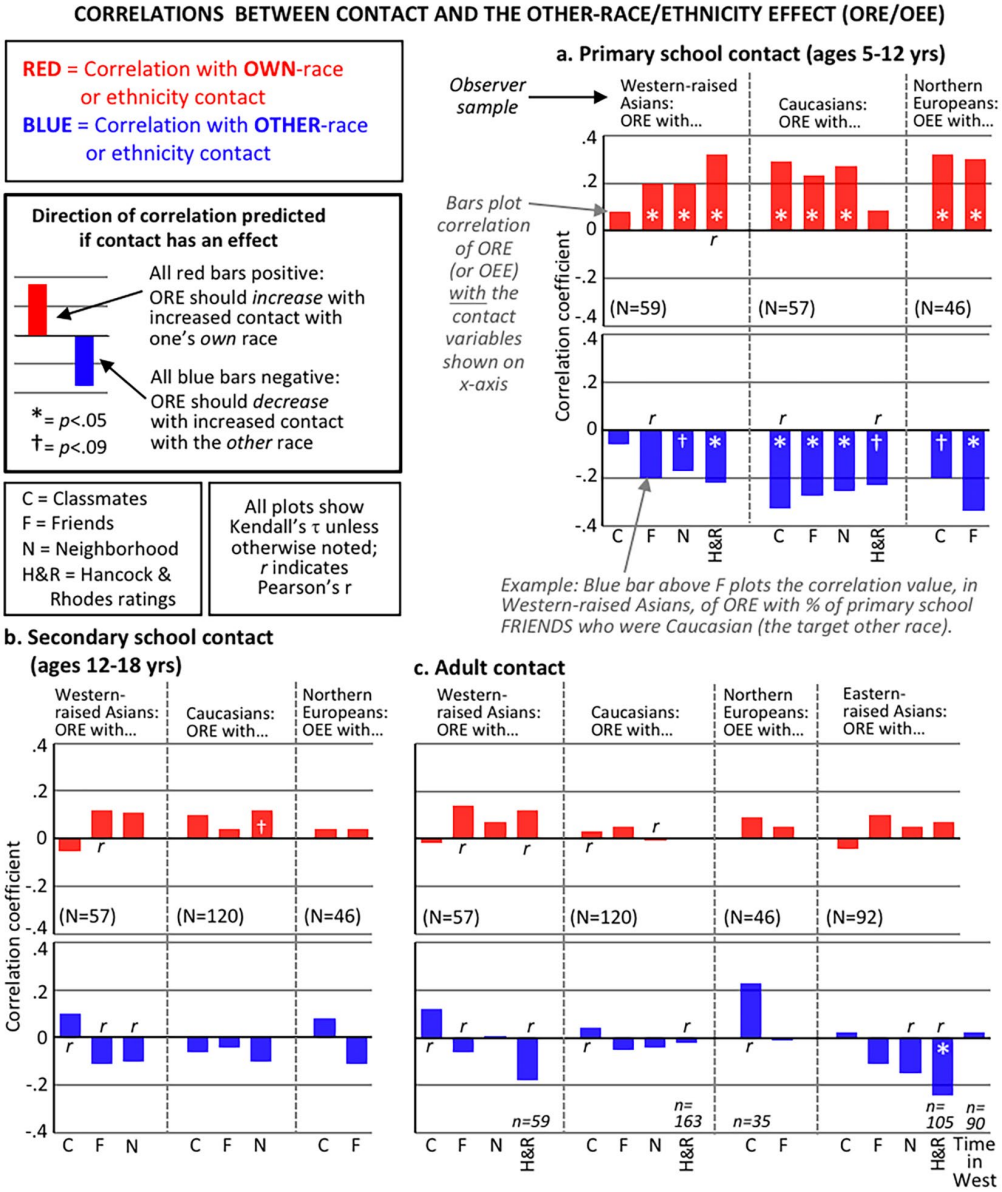
Results: Other-race face recognition is more plastic in childhood than in adulthood. Each bar shows the correlation, within one of the four observer samples, of individuals’ ORE/OEE difference score (Fig. 2) with various measures of their contact (Fig. 3) listed on x-axis, separated by contact with people of their own- race/ethnicity (red bars) and contact with the relevant other-race/ethnicity (blue bars). Results are shown for: (**a**). Contact experienced in primary school (showing 20/20 correlations in predicted direction with 13/20 significant at *p* < 0.05 uncorrected; *p* < 0.000001 for this total evidence pattern, computed using Monte Carlo simulations that account for the intercorrelation between different contact measures; see Methods); (**b**). Secondary school (*p* = 0.063 for total evidence pattern); and (**c**). As adults (*p* = 0.119 for total evidence pattern). All observers were tested as adults, and self-reported contact at previous or current life stages. For explanations of tau vs r (determined by whether contact distribution is skewed), sample size, missing data, see Method, Tables S1–4.

Figure 4 shows the key results. Tables S2–4, S6–9 list distributional information for all variables, plus reliability values, exact correlations, and exact *p* values. Preliminary data checks confirmed: ORE and OEE difference scores were normally distributed (Table S8); and the input CFMT tests had, as in previous studies^28,29^, a wide range of individual differences in performance with no problems concerning ceiling effects (for own-group tests) or floor effects (for other-group tests). Contact distributions were often skewed, particularly in Primary school; Tables S2–4 give details and rationale for reporting Kendall’s tau or Pearson’s r based on whether the contact distribution showed significant skew. Note the Eastern-raised Asian group were not analysed for school-aged contact due to an almost complete lack of range in scores (median of 0% school exposure to Caucasians; see Table S1).

The hypothesis under consideration is that amount of contact with a particular face type drives relative recognition performance. This predicts effects of contact with other-group people but, importantly, also effects of contact with *own*-group people. If face recognition remains plastic at a given age, the predictions are that: (a) the size of the ORE (or OEE) should *decrease* with increasing contact with people of the relevant *other*-race (or ethnicity), producing a *negative* correlation (i.e., bars colour-coded blue in Fig. 4 should all be *below* zero); and (b) at the same time, the size of the ORE (or OEE) should *increase* with increasing contact with *own*-race (or ethnicity) people, producing a *positive* correlation (bars colour-coded red should all be *above* zero; this is because greater own-race experience should improve own-race recognition relative to other-race recognition).

Results in Fig. 4 clearly support a critical period, in which plasticity in response to contact with other- versus own-race/ethnicity people is greatest in the first part of childhood (primary school) and then decreases with age. The size of the ORE or OEE correlated with contact the observer reported having experienced when they were in primary school (5–12 years), but not with the contact they had experienced when in secondary school (12–18 years), nor with their current contact as an adult. This conclusion was replicated across all four independent samples.

For contact obtained during primary school (5–12 years), Fig. 4a shows data closely matched the predicted pattern of correlations. Across the 20 correlation bars, the total evidence pattern was: 20/20 in the predicted direction for a contact effect (i.e., the ORE/OEE increased with greater own-group contact and decreased with greater other-group contact), with most of these (13/20) significantly different from zero at *p* < 0.05 uncorrected (two-tailed tests), and with a further 3 approaching significance (at *p* < 0.09). Computational simulations (see Methods, designed to deal with skewed distributions and the presence of intercorrelations between contact measures) were used to determine a condition-wise Type I error rate for primary school contact — that is, the chance of obtaining this total evidence pattern across the 20 correlations if, in fact, there was no underlying relationship between primary school contact and the ORE/OEE. Results revealed a highly significant association between primary school contact and the ORE/OEE, at *p* < 0.000001.

For contact obtained during secondary school (12–18 years), these effects disappeared or weakened considerably. Of the 16 contact correlations in Fig. 4b, three were in the reverse-to-predicted direction, none were significant, and only one approached significance. The condition-wise Type I error rate for this evidence pattern was *p* = 0.063, indicating no significant association between secondary school contact and the ORE/OEE (i.e., *p* > 0.05; although note a small-but-real relationship cannot be entirely ruled out at *p* = 0.063, see Discussion).

For contact obtained in recent life as an adult, results indicate no contact effect. Of the 29 correlations in Fig. 4c, 10 were in the reverse-to-predicted direction, and only one is significant in the predicted direction (at *p* < 0.05 uncorrected); note the other substantial looking correlation (*r* > 0.2 in the OEE plot) is in the reverse-to-predicted direction. Also note that for Eastern-raised Asians who moved to the West as adults for university study, the lack of any correlation with Time in the West was obtained despite a substantial range from 1 month to 5 years since arriving in Australia (Fig. S1 for scatterplot). The condition-wise Type I error rate for the total evidence pattern was *p* = 0.119, indicating no significant association between adult contact and the ORE/OEE.

Some readers may also be interested in results for the *raw* scores of other-race/ethnicity memory with other-race contact. In contrast to the ORE/OEE difference scores, raw CFMT memory scores offer less statistical power because they fail to remove variance associated with general cognitive abilities (e.g., general visual memory ability). Despite this, results for raw scores in Tables S10–S12 show the same broad pattern as described above. For primary school contact, correlations with CFMT-other (Table S10), while smaller than with ORE/OEE (Fig. 4) due to the unremoved variance, still show 8/10 in the predicted direction, one of which is significant at *p* < 0.05, and one approaching at *p* < 0.1. And, for secondary and adult contact, there is again no evidence of any contact effects: for secondary, 4/6 in predicted direction (none approaching significance, all *p*s > 0.25); for adult, only 7/15 in predicted direction (none approaching significance, all *p*s > 0.25).

### Quality versus quantity of contact

Figure 4 shows that, while the age of contact was critical, the type of contact did not matter. There was no evidence of any consistent differences between contact with friends versus classmates versus neighbours, providing no support for the theory that “high-quality” contact might be more effective in reducing the ORE than mere exposure.

### Can we infer causation from our childhood contact correlations?

Can we infer from Fig. 4 that primary school contact is a *causal* factor in the chain of events that result in the adult ORE? Unusually for correlational data, a conclusion of causation seems reasonable, due to the inherent longitudinal nature of the relationship. It is entirely plausible that *current* (adult) other-race effects were caused by the contact obtained *in a preceding time-period* (i.e., 10 or more years earlier when the participant was at primary school). Certainly, it is not possible for a participant’s adult ORE to have caused a change in their early-life contact. It is also unlikely that a participant’s adult ORE caused relevant errors in their *recollection* of their earlier-life contact (noting we used retrospective self-report): the oldest recollections (i.e., of primary school contact) would be expected to be the most noisy, and yet it was only these that correlated with the ORE/OEE (Fig. 4).

### Lack of secondary and adult correlation is valid, and validation of self-reports of contact via correlation with prejudice

The conclusion of enhanced plasticity in childhood relies as much on the absence of correlations at later ages as it does on the presence of correlations in primary school. It is thus important to establish that the lack of correlations of the ORE/OEE with secondary school and adult contact in Fig. 4 does not reflect uninteresting methodological problems.

First, correlations in Fig. 4 are not Bonferroni corrected, to avoid inflating Type II error for conditions where the prediction is of *no* or weak correlation.

Second, the lack of secondary and adult correlations in Fig. 4 cannot be attributed to problems with the *range* of the contact variables. It was *primary* school contact that had some problems with restriction of range (Method); yet despite this issue, and the resulting likely underestimation of the true primary-school correlation strength, significant correlations with ORE/OEE emerged. The contact range in secondary school and adulthood was larger than that for primary school (Tables S2–4), clearly giving sufficient room for correlations with later-age contact to have been revealed, had these existed. Yet correlations with ORE/OEE did not emerge at these later developmental stages.

Third, the lack of secondary and adult correlations cannot be attributed to the use of self-reported contact. Figure S2 shows that secondary and adult contact scores successfully produced significant correlations with prejudice (willingness to marry an other-race person) in the predicted direction. This replicates the standard finding (e.g.^21–23^) that increased contact is associated with reduced prejudice, thus validating the self-report measures and also confirming their reliability by showing they are not too noisy to produce correlations.

### Can the other-race effect be completely avoided?

**Figure 5:**
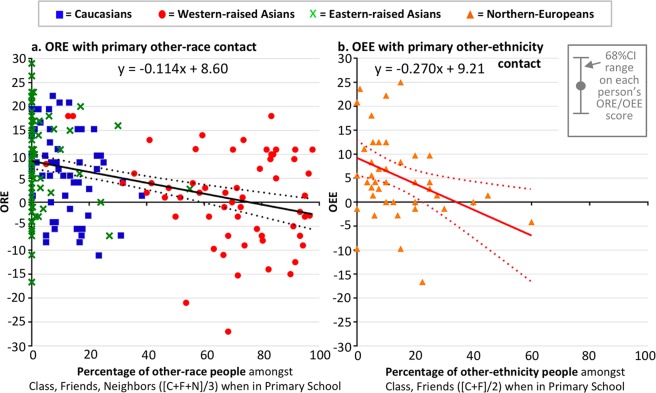
Sufficient primary school contact can completely eliminate other-race and other-ethnicity effects. Plots show individual-participant scores for (**a**). ORE and (**b**). OEE. Solid trendlines (with equations) show line of best fit. Dotted lines are 95%CI on the trendline. Error bar at top right indicates approximate 68%CI on each individual participant’s ORE/OEE score (Table S6 for exact values); this illustrates that much of the variability in individual scores represents measurement error rather than variance that needs to be explained theoretically.

Results so far indicate the ORE is reduced with increasing other-race contact, as long as this contact is obtained before approximately 12 years of age. This raises the question of whether it could be avoided altogether with enough contact in this early developmental stage. Figure 5a plots the size of the ORE as a function of primary school contact (total contact, averaging Classmates, Friends, and Neighbours), combining all samples to most fully map out the effects of differing contact levels (noting that the three samples differed in mean contact). Results indicate sufficient primary school contact can completely remove the ORE, and also the OEE (Fig. 5b).

### Our participants’ ORE is not of social-attitude origin

In our particular cultural setting — Asians and Caucasians tested in Australia with similar socioeconomic status — we have previously shown the ORE has no social-attitudinal component (e.g., no influence of motivation-to-individuate instructions^7^). Further supporting this conclusion, we found here that prejudice (willingness to marry a person of the other-race) did not predict the ORE (Fig. S3).

## Discussion

Our study provides the first evidence that plasticity of face recognition for different face types is greater in childhood than in adulthood. Our results demonstrate sensitivity to natural social contact during primary school ages (5–12 years) and, equally important, *lack* of sensitivity to such contact at later ages (particularly adulthood). These findings were replicated across four independent participant samples. The replications also demonstrated generalisation across multiple situations that included: people raised in countries where their own race is the majority; people raised in countries where their own race is the minority; two races of observers; and faces with larger morphological differences between categories (differing in *race*) and faces with smaller morphological differences between categories (differing in within-race *ethnicity*).

Our findings have important implications for a range of theoretical and practical issues. We describe these below, after first dealing with a methodological issue concerning the possibility that our results are limited due to our reliance on retrospective report.

### Measuring contact via retrospective report

Where the aim is to assess the effects of natural, social contact at different stages of development, methodological options are limited. Longitudinal approaches are not practical; that is, it is not feasible to track hundreds or thousands of children over, say, 15 years (5 yrs old to 20 yrs old), recording their number of other-race classmates and friends every year, and hoping that the final sample will contain primary school contact that is sufficiently dissociated from later-stage contact to allow age-of-contact effects on adult ORE to be assessed. Thus, prior-life contact was necessarily measured via retrospective self-report.

Two issues concerning these self-report measures then arise: reliability and validity. Concerning *reliability* (i.e., level of noise in the measurements), it is clear that reliability was adequate. First, all contact measures were able to reveal correlations with other variables (ORE/OEE and/or prejudice). Second, we would expect the oldest recollections of contact to be the most noisy (i.e., primary school), yet even these were quite capable of revealing significant correlations. Third, for the Hancock and Rhodes^8^ contact questionnaire measure, it is possible to calculate internal reliability because there are multiple items on the scale, and this was high (Cronbach’s alphas = 0.74-0.93 for both primary school and high school; Table S9). Fourth, our Time-in-West measure for Western-raised Asians should have essentially no noise (i.e., international students are well aware of when they first arrived in Australia).

Concerning *validity*, results confirmed our contact measures *correlated with prejudice in the predicted direction*, i.e., they reproduced the standard finding that greater other-race contact is associated with reduced prejudice. Additionally, further evidence arguing that contact scores were unlikely to be biased — for example, by participants somehow *estimating* their previous number of other-race classmates or friends based on their *current* level of fluency with other-race faces — are the facts that: time periods for reporting contact were chosen to be easily separable in memory; the time periods were not too far in the past (e.g., there seems no reason why 18–22 year olds would fail to directly remember the make-up of their school classes, or who their friends were in school); and, most compellingly, any use of estimation based on current fluency with other-race faces, rather than actual recollection, should have biased *all* contact measures yet our data show other-race recognition ability was associated only with primary and not secondary or adult contact scores.

### Resolving the longstanding conflict concerning contact effects: The importance of asking about the age at which contact occurred

The question of whether other-race face recognition ability is influenced by contact is core to many theories of ORE origin. Yet, previous results vary dramatically across studies (e.g.^7–9^, versus^1,10–13^). Our results offer resolution of this longstanding conflict, arguing the confusion arises due the presence of a crucial unmeasured variable, namely the *age at which the contact occurred*.

Additionally, our results fail to support the proposal^5^ that the conflicting findings might be due to confounds with *quality* of the contact. Like several previous studies^8,26,27^, we find no support for the idea that “high-quality contact” (as assessed here by number of other-race friends, and also the Hancock and Rhodes^8^ questionnaire) is any more useful in reducing the ORE than mere exposure. Importantly, however, we extend these previous findings to show the conclusion holds for *all ages* at which contact is experienced: high-quality contact and mere exposure are equally *effective* in childhood, and equally *in*effective in adulthood.

### A critical period for face recognition

Comparing our age-of-contact results to previous literature on face plasticity, the question of whether plasticity might reduce as children grow into adults has recently been recognised as being of major theoretical importance^15,30^. For other-race faces, while it is established that post-infancy childhood plasticity exists (the ORE can be removed by childhood adoption to an other-race country^18,19^) and that there is some flexibility remaining in adulthood when explicit training is provided (trial-by-trial feedback across several thousand trials can produce some improvement in recognising other-race faces^31^), it has been difficult to compare the *relative* degree of plasticity between developmental stages, particularly for natural social exposure which is likely to induce implicit, non-effortful, learning and does not involve explicit training. This issue has arisen partly because previous studies of contact have not recorded contact at different ages. More fundamentally, however, it has arisen because many demographic settings around the world are unsuitable for addressing the age-of-contact question, because contact remains stable across a participant’s lifetime. A key aspect of the present study was the access to many participants for whom the balance of face types to which they are exposed has changed between childhood and adulthood; this allowed us to examine, and reveal differential effects of, contact at different developmental stages.

Our results clearly demonstrate that other-race face recognition is sensitive to contact in primary school, before the age of approximately 12 years. Equally clearly, our results demonstrate that other-race face recognition is *not* sensitive to contact as an adult after 18 years (even with up to 5 years exposure in a country where the other-race forms the majority of people, Fig. S1). This combination demonstrates a critical period during childhood for easy acquisition of face recognition ability via natural, social exposure in everyday life. It also implies that, by adulthood, any improvement in other-race face coding ability will be difficult and require a different type of learning (e.g., explicit training-with-feedback^31^).

### When does the critical period for face recognition end? Contact in the teenage years

Our data leave open exactly when the sensitive window for natural social contact closes. It is possible that full closure does not occur until sometime during the teenage years. Certainly, the effect of teenage contact on the ORE/OEE was much weaker than the effect of pre-teen contact (Fig. 4), particularly noting that the pre-teen contact correlations are expected to be underestimates of the true strength due to the skewed contact distributions for primary school. However, a small teenage-contact effect cannot be completely ruled out. With N = 223, the total evidence for a contact effect in Fig. 4 sat at p = 0.063. Thus, while we can conclude that by far the greatest sensitivity to natural social contact is prior to 12 years of age, it is also possible that some minor sensitivity remains during the teenage years that might be statistically significant with very large sample sizes (as, indeed, has recently been demonstrated with extremely large sample sizes for language^16^).

### Parallels between faces and language across the full developmental course

Turning to the developmental plasticity of face recognition versus language, our results demonstrate strong similarity between these domains. Putting our own results together with previously-established findings, we conclude face recognition now mirrors language across the *full* developmental course shown in Fig. 1 (Boxes a–d). In both domains, this pattern can be summarised as: initial broad ability (Box a, e.g., early-infancy discrimination of individual faces for races never experienced); perceptual narrowing across infancy (Box b, loss of this initial discrimination ability without post-birth exposure, e.g., Chinese babies’ loss of early ability to individuate Caucasian faces)^32^; retained plasticity to easily reverse perceptual narrowing in childhood (Box c); and greatly reduced plasticity in teenage and adult years (Box d).

Note we are not suggesting the time course of sensitive periods is necessarily *exactly* the same in face recognition as in language; indeed evidence suggests different durations for different aspects of language within infancy^14^. However, we argue the face recognition system and the language system share a key property of a period of enhanced plasticity, in response to natural real-world experience, earlier in development compared to the adult state.

### What ties faces and language together? A shared role in social communication

Why does this similarity between face recognition and language occur? Answering this question requires knowing, first, whether the developmental pattern in Fig. 1 is limited to these domains, or is a general phenomenon that occurs for all visual and auditory stimuli. Evidence argues against domain generality. Domain generality would predict that every box in the Fig. 1 developmental pattern would be found for *any* stimulus type. Yet, clear violations exist. First, concerning Box a, young-infants’ ability to tell apart individual exemplars of the category even with no previous experience of the category (which is found for phonemes and upright faces) does not occur for upside down faces^33^, nor for baby strollers^34^ (babies must be explicitly *trained* with feedback to tell apart individual strollers^34^). Further, like faces, humans have extensive everyday visual exposure to *hands* throughout both infancy (Box b) and childhood (Box c), yet this natural social exposure fails to result in adults developing good ability to discriminate other people from their hands^35^. Finally, we note that reading—an evolutionarily recent cultural invention which, unlike spoken language, needs to be explicitly taught—produces similar cortical changes regardless of whether it is learned in childhood or as an adult^36^.

We suggest the developmental similarity between faces and language arises from their *shared role in social communication*. Successful communication between people requires perceiving *who* (faces) is signalling *what* (e.g., via language) to *whom* (faces again). Critically, all stimuli related to these abilities have been found to follow the infant developmental pattern in Fig. 1. Specifically, innate early-infancy discrimination (Box a) followed by perceptual narrowing (Box b) has been demonstrated for: phonemes^14^; speech tones^14^; static faces^14^; silent talking faces^14^; visual sign language^14^; discrimination of individuals by their voice^37^; and cross-modal matching of individuals’ voices to their faces^14^. The strong theoretical importance of these infant developmental links has previously been noted^14,38^. Our present results extend the similarity between face processing and language beyond infancy, to across childhood and adulthood (i.e., Boxes c and d in Fig. 1).

What might help to maintain similarity of development over such a long time-frame? We suggest several factors relevant to social communication might contribute. First, faces and speech share a *location in physical space*; that is, the voice emerges from part of the face, and so attending to speakers and attending to facial information require attending to the same physical location. Second, like faces, humans use voices as a key way to *uniquely identify individual people*^39^. Third, again like faces, humans use speech (e.g., notably accent), as a major way we *categorise people into social groups*^40^.

### Implications for theories of the ORE

Our findings also have important implications for theories of the ORE. First, our results support a core role for perceptual experience in the origin of the ORE (also see, for example^7–9,41,42^). This rules out pure social motivational theories, that is, any theory in which the ORE is attributed *only* to social outgrouping, prejudice (explicit or implicit), attitudinal bias, lack of effort, or attention to race-category facial characteristics (e.g., light skin tone, thin lips, and prominent-noses-in-profile for Caucasians) rather than within-race individuating characteristics. Instead, our results argue that only theories that include at least some role for perceptual experience are in the running.

Second, our results argue that all previous theories involving perceptual experience (e.g.^5–7^) require a major modification, namely that it is not lack of perceptual experience per se that results in an ORE, but specifically a lack of *childhood* perceptual experience.

Third and more broadly, evidence across the literature overall favours a theoretical approach in which the total size of the ORE may be a *sum of* social-motivation and childhood experience contributions; this would be similar to our Wan *et al*.^7^ dual-factors model, with the modification that the experience-only route becomes a route for *childhood* perceptual experience. In our present cultural setting — namely Asians and Caucasians in a country where these groups are of equal socioeconomic status — findings argue for *no* social-motivational component to the ORE(^7,13,43^; plus present result that prejudice did not predict the ORE), meaning that *only* childhood perceptual experience will contribute. However, in a different cultural setting — USA White observers looking at African-American faces (i.e., groups with large differences in socioeconomic status) — a social-motivation contribution to the ORE has been reported^6^. In that cultural context, we argue the ORE will include contributions from both social-motivation and childhood experience, meaning that, although the ORE would still be *reduced* by childhood (and not adult) experience, *removing* the ORE altogether may require also increasing motivation to individuate the other-race faces.

### Practical implications: Encourage early-life exposure, or require difficult, time-consuming training as an adult

In many countries in the modern world, successful social interaction requires being able to recognise any individual, regardless of race^44,45^. As perceivers, how can we best ensure that we are able to successfully recognise other-race people?

Our results imply that, as with language, by far the easiest method is to obtain everyday exposure as a child in natural social environments. Our results confirm that natural exposure as a young child can produce excellent recognition of other-race individuals. Indeed, with enough exposure within the course of everyday social experience a Caucasian child can become effectively a “native recogniser” of Chinese faces (and vice versa), just as, in language, a young English-speaking child who moves to France can become a native speaker of French.

Equally, however, our results imply that the developmental window for this easy acquisition closes (or at least narrows very substantially) by approximately 12 years of age. Where childhood exposure was low, overcoming poor other-race recognition as an adult is not easy. Importantly, our results show it cannot be achieved simply via increasing everyday exposure, even “high quality” exposure to other-race friends. Instead, as with second-language learning, other-race face learning as an adult is likely to require explicit, time-intensive, and expensive training procedures^31^.

Thus, to reduce the many negative consequences of poor other-race face recognition — which include implicit racism, wrongful convictions, social interaction difficulties and security failings — other-race experience in childhood should be encouraged.

## Methods

### Terminology: Definition of race and ethnicity

For face stimuli and participants, we use the term *race* to refer to the relatively large physical differences in faces with ancestry from different continents or major subcontinents, specifically focussing here on Asian and Caucasian (meaning European-heritage). All participants reported being of a single race only; anyone indicating mixed-race heritage was excluded. The term ‘Caucasian’ was used in preference to alternatives (‘White’, ‘European’) because it is the standard term in Australian public discourse (e.g., used by police when describing a wanted offender). The term ‘Asian’ here means East Asian or South-East Asian (e.g., from China, or Singapore), not South Asian (i.e., not from India, Pakistan).

We use the term *ethnicity* to refer to the smaller within-continent physical differences, specifically focusing here on the largest distinction within Europeans, namely those from Northern Europe including Britain, versus those of more Southern European appearance.

### Participant groups

Participants (total N = 373; mean age = 21.1 years, SD = 3.9) were recruited from Australian universities. For the ORE, we tested three samples. *Caucasian observers* were all born and raised in Western majority-Caucasian countries, and were studying at the Australian National University (ANU). *Asian observers (Eastern-raised)* were all born and raised in Asia, had moved to Australia for university study when aged 17 years or older (mean time living in Australia = 16.1 months, SD = 13.3, range = 1–61), and were studying at ANU. *Asian observers (Western-raised)* were born and raised in Western majority-Caucasian countries (mean time spent in Asia holidaying or visiting relatives across their life to date was 5.0 months, SD = 19.0), and were studying at ANU or University of Western Australia. In all cases, the own-race stimuli were also well matched to the sample group on *within*-race ethnicity (i.e., Asian participants and faces were primarily Chinese-heritage; Caucasian participants were primarily Northern-European heritage, which is why the primarily Northern-European-heritage face test was chosen for the own-race condition).

For the OEE, participants were *Northern-European-heritage Caucasian observers*, born and raised in Australia (or other Western country). They were specifically of British origin, reporting 100% British Isles ancestry, i.e., all known ancestors originating from England, Ireland, Scotland, or Wales; this is the most common ethnic group in Australia. They were tested at ANU. Table S5 gives sex and age for samples in Fig. 4, plus remuneration details.

We obtained the largest sample sizes we could by collating CFMT data across several studies conducted partly for other purposes (with previous publications analysing different aspects of the data than the present article; Supplementary Information Appendix 1 provides details). We analysed all participants from those studies who met our present inclusion criteria (i.e., race/ethnicity criteria, plus having scores on the contact questionnaire/s as well as on both the own- and other-group CFMT tests).

All experimental protocols were approved by the ANU Human Research Ethics Committee, and the studies were carried out in accordance with guidelines of the National Health and Medical Research Council of Australia. Informed consent was obtained from all participants.

### Face recognition tasks and calculation of individual’s ORE/OEE score

All face recognition tasks used the standard Cambridge Face Memory Test (CFMT) format and procedure^20^ (details in Supplementary Information Appendix 2). Briefly, each test requires learning 6 target faces, and later discriminating these from other non-target faces in 72 three-alternative-forced-choice trials, each showing the target plus two distractors. To ensure *face* recognition ability is measured, rather than recognition of a particular photograph, each target is learned and tested in multiple images varying in view and lighting, and hair and clothing are excluded from the images. As is usual in ORE studies, no mention was made to participants of race or ethnicity before testing. A major reason for choosing the CFMT format is that it produces good reliability^28,29^ (Cronbach’s alpha in present samples 0.81-0.89; Table S6); this attribute is important when analysing correlations.

We used three available variants of the CFMT. For the Asian face stimuli, we used the CFMT-Chinese^24^. For the “Northern-European” Caucasian face stimuli, we used the CFMT-Australian (which contains faces of primarily British ancestry)^25^; for the “Southern-European” Caucasian face stimuli, we used the CFMT-original^20^. Note these latter tests do not contain faces solely of one type of European heritage, but they differ in facial appearance on average (Fig. 2).

Each participant’s ORE or OEE was calculated as the difference between their own-group and other-group recognition accuracy (% correct). This procedure is standard in studies testing contact correlations (e.g.^6,8^). It is used because own-group face recognition ability varies substantially across individuals^28^, so using a difference score assesses each individual’s other-group face recognition relative to their own “best case” ability.

### Questionnaire measures: Contact and willingness-to-marry

Questionnaires were administered at the end of the session, after the face tests. Full wording of contact measures (Fig. 3) is provided in Supplementary Information Appendices 3–5.

Our marriage attitude item was designed to pick up even subtle differences in prejudice. Participants were asked about their willingness to marry an other-race person (Supplementary Information Appendix 6 for full wording). The scale ranged from 1 = “I would consider only someone of my own race” to 9 = “I would consider only someone of another race”. Mean response was mid-way between 3 = “I would consider people from any race more-or-less equally, although with a small preference for my own race” and 4 = “I would consider people from any race equally as long as they were all equally nice, although probably it would be a bit easier if they were my own race” for the two groups raised in majority own-race countries, and midway between 4 and 5 “I would be completely neutral as to the person’s race” for the Western-raised Asians. Range was good for analysing correlations (see Table S7).

### Explanation of sample sizes: Cases of insufficient range of contact to analyse correlations

We were sometimes unable to validly analyse correlations with contact due to insufficient range of contact with the other race in the relevant life stage. For Eastern-raised Asians, for both primary school and secondary school, the median number of Caucasian classmates reported by this group was 0%; thus, this group was analysed only for adult contact. For Western-raised Caucasians, lack of range in contact with Asians led to a smaller sample size for primary than for secondary or adult contact. We first began this project in 2009 when we conducted a preliminary test of contact range with Asians: results showed the median number of Asian primary school classmates reported was only 5%. We were aware, however, that the number of Asians in the population of our testing city (Canberra) had been increasing over time and we expected that primary school contact in our university adult samples would eventually produce a testing-year with sufficient range to analyse contact correlations. Setting an inclusion criterion of a minimum of 10% for the classmates median—on the grounds that anything less than this clearly indicated insufficient range (i.e., nonsignificant correlations could not be taken as any evidence of lack of relationship)—we tested in 2012, 2013 and 2015. As shown in Table S1, it took until 2015 for sufficient demographic change to occur for analysis of primary contact to become feasible (i.e., reaching the 10% criterion). This explains the smaller n for primary (n = 57, 2015 only) than for secondary and adult correlations (N = 120) because for the latter all testing years (2012, 2013, and 2015) met the inclusion criterion. For more minor variations in sample size within Fig. 4, see explanation of missing data in Supplementary Information Appendix 7.

### Preliminary data checks

Tables S2–4 and S6–9 present distributional and internal reliability information for all variables. Before reporting correlations, we checked all scatterplots for bivariate outliers: we found only one problematic outlier, and replaced this score with the next-highest score on each variable. Scatterplots showed no evidence of nonlinear relationships.

### Dissociation of contact across different ages: Demographics and immigration history

It is crucial to our design that contact did not simply stay stable over individuals’ lifetimes. To achieve dissociation requires a sample containing many participants for whom the balance of face types they are exposed to has changed between childhood and adulthood. For example, a Caucasian-observer sample might contain: some participants who saw more Asians as adults than they saw as children; some participants who saw a similar proportion of Asians at each age; and even some participants who saw more Asians as children than as adults, thus producing a low correlation between childhood and adult contact within the sample. In many world locations, such a sample would be difficult to obtain because contact in that location tends to stay stable across the lifetime for all or most participants. Here, we were able to obtain suitable samples due to specific aspects of demographics in Australia, arising from a combination of: Australia’s migration history; differences between city and country regions; selective government schools; and university intakes (Supplementary Information Appendix 8 for details). Indicating the needed level of dissociation of contact across age, correlations between all life stages was low-to-medium rather than high (Table S13).

### Monte Carlo simulations to compute overall Type I error rate for each age-of-contact

Condition-wise *p*-values for each age-of-contact were calculated using Monte Carlo simulations. We estimated the overall Type I error rate from the total pattern of correlations between contact and the ORE/OEE, separately for each of the three age-of-contact conditions. Specifically, we compared our total pattern of evidence concerning a contact effect for that age-of-contact, to an estimate of the probability of obtaining such evidence if there were no true correlation between contact-at-that-age and the ORE/OEE.

For example, for Primary School contact, our total pattern of evidence across all 20 correlation values plotted in Fig. 4a was as follows: all 20 correlations were in the predicted direction (i.e., the direction predicted by the contact hypothesis), with 13 of these significant on individual-correlation tests at *p* < 0.05 (two-tailed, uncorrected for multiple comparisons), and a further 3 approaching significance at *p* < 0.09. Our aim was to determine the chance of obtaining a total pattern of evidence this strong, or stronger, if in reality there was no true relationship between primary school contact and the ORE/OEE.

To do so, it was important to take into account two key statistical features of the data. First, many of the contact measures were not normally distributed (indeed some were quite highly skewed). Second, some subsets of correlation values were *independent* — specifically, values from the 4 different participant samples — while others were *dependent*: within each participant sample, there were intercorrelations between the different contact variables (Tables S14–16).

Using Monte-Carlo simulations as the method of combining evidence across the different measures allowed us to account for these two key features. To maintain the actual distribution shapes, and the actual degree of intercorrelation between some contact measures (and lack of it between others), we used the original contact scores exactly as they were (i.e., correctly lined up with the participant codes). We also used the actual ORE/OEE scores for a given participant sample, but, crucially, on each run we randomised the assignment of the ORE/OEE scores to participant codes so that there was, *a priori*, no true relationship between contact and the ORE/OEE. Correlations were calculated using tau (for skewed contact variables) or Pearson’s *r* (for non-skewed contact variables) as specified in Fig. 4a. We then repeated this procedure for 1 million runs (using software *R*), each with a different randomisation of the ORE/OEE scores, and counted the number of runs which produced evidence as strong as, or stronger than, our actual results (Table S17).

Importantly, this procedure produces a more conservative p-value than say, an analysis which ignored the intercorrelations between contact measures. To illustrate with a concrete example, consider Secondary School contact and just the *direction* of the correlations for simplicity of argument. (Note our real analysis also coded for whether correlations were significant or not). For Secondary contact, we obtained 13/16 correlations in the predicted direction. The probability of getting 13/16 independent correlations in the right direction is *p* = 0.0085 (by the binomial distribution). Thus, had we ignored the intercorrelations between contact measures, we would have wrongly concluded the Secondary contact effect was significant. The true evidence for an effect is weaker, with the exact amount weaker being dependent on the exact degree of intercorrelation between all the different contact measures. Our Monte Carlo simulation, which maintains the specific intercorrelation values, showed that the *p*-value for 13/16 in the right direction in our secondary data set was only *p* = 0.0878 (Supplementary Table S17), i.e., not significant.

## Supplementary information

McKone_face_race_critical_period_Supplementary_Info

Dataset 1

Dataset 2

Dataset 3

## Data Availability

**Data**: De-identified data files can be accessed as Supplementary Excel files (3 separate files, one for each age of contact). **Materials**: CFMT tests available from EM; questionnaire measures provided in Supplementary Information.

## References

[1] Meissner CA, Brigham JC (2001). Thirty years of investigating the own-race bias in memory for faces. Psychol., Pub. Policy, & Law.

[2] Meissner CA, Susa KJ, Ross AB (2013). Can I see your passport please? Perceptual discrimination of own- and other-race faces. Vis. Cog..

[3] Qian MK (2017). Perceptual individuation training (but not mere exposure) reduces implicit racial bias in preschool children. Dev. Psychol..

[4] Scheck, B., Neufeld, P. & Dwyer, J. *Actual innocence: When justice goes wrong and how to make it right*. New edn, (New American Library, 2003).

[5] Valentine T, Lewis MB, Hills PJ (2016). Face-space: A unifying concept in face recognition research. Quarterly J. Exp. Psychol..

[6] Hugenberg K, Young SG, Bernstein MJ, Sacco DF (2010). The categorization-individuation model: An integrative account of the other-race recognition deficit. Psychol. Rev..

[7] Wan L, Crookes K, Reynolds KJ, Irons JL, McKone E (2015). A cultural setting where the other-race effect on face recognition has no social–motivational component and derives entirely from lifetime perceptual experience. Cognition.

[8] Hancock KJ, Rhodes G (2008). Contact, configural coding and the other-race effect in face recognition. Brit. J. Psychol..

[9] Zhao M, Hayward WG, Bülthoff I (2014). Holistic processing, contact, and the other-race effect in face recognition. Vis. Res..

[10] Brigham J, Barkowitz P (1978). Do “they all look alike?” The effect of race, sex, experience, and attitudes on the ability to recognize faces. J. Appl. Soc. Psychol..

[11] Michel C, Caldara R, Rossion B (2006). Same-race faces are perceived more holistically than other-race faces. Vis. Cog..

[12] Ng W-J, Lindsay RCL (1994). Cross-race facial recognition: Failure of the contact hypothesis. J. Cross-Cultural Psychol..

[13] Tullis JG, Benjamin AS, Liu X (2014). Self-pacing study of faces of different races: metacognitive control over study does not eliminate the cross-race recognition effect. Mem. & Cog..

[14] Maurer D, Werker JF (2014). Perceptual narrowing during infancy: A comparison of language and faces. Dev. Psychobiol..

[15] McKone E, Crookes K, Jeffery L, Dilks D (2012). A critical review of the development of face recognition: Experience is less important than previously believed. Cog. Neuropsychol..

[16] Hartshorne JK, Tenenbaum JB, Pinker S (2018). A critical period for second language acquisition: Evidence from 2/3 million English speakers. Cognition.

[17] Norrman G, Bylund E (2016). The irreversibility of sensitive period effects in language development: evidence from second language acquisition in international adoptees. Dev. Sci..

[18] de Heering A, de Liedekerke C, Deboni M, Rossion B (2010). The role of experience during childhood in shaping the other-race effect. Dev. Sci..

[19] Sangrigoli S, Pallier C, Argenti A-M, Ventureyra VAG, de Schonen S (2005). Reversibility of the other-race effect in face recognition during childhood. Psychol. Sci..

[20] Duchaine BC, Nakayama K (2006). The Cambridge face memory test: Results for neurologically intact individuals and an investigation of its validity using inverted face stimuli and prosopagnosic participants. Neuropsychologia.

[21] Brigham JC (1993). College students’ racial attitudes. J. Appl. Soc. Psychol..

[22] Brigham, J. C., Bennett, L. B., Meissner, C. A. & Mitchell, T. L. In *The Selected Works of Christian A*. *Meissner*, *Ph*.*D*. 28 (2007).

[23] Slone AE, Brigham JC, Meissner CA (2000). Social and cognitive factors affecting the own-race bias in Whites. Basic & Appl. Soc. Psychol..

[24] McKone E (2012). A robust method of measuring other-race and other-ethnicity effects: The Cambridge Face Memory Test format. PLoS One.

[25] McKone E (2011). Face ethnicity and measurement reliability affect face recognition performance in developmental prosopagnosia: Evidence from the Cambridge Face Memory Test - Australian. Cog. Neuropsychol..

[26] Carroo AW (1987). Recognition of faces as a function of race, attitudes, and reported cross-racial friendships. Percept. & Motor Skills.

[27] Walker PM, Hewstone M (2006). A perceptual discrimination investigation of the own-race effect and intergroup experience. Appl. Cog. Psychol..

[28] Bowles DC (2009). Diagnosing prosopagnosia: Effects of ageing, sex, and participant-stimulus ethnic match on the Cambridge Face Memory Test and Cambridge Face Perception. Test. Cog. Neuropsychol..

[29] Wilmer JB (2010). Human face recognition ability is specific and highly heritable. Proceed. Nat. Acad. Sci..

[30] Macchi Cassia V, Kuefner D, Picozzi M, Vescovo E (2009). Early experience predicts later plasticity for face processing: Evidence for the reactivation of dormant effects. Psychol. Sci..

[31] Tanaka JW, Heptonstall B, Hagen S (2013). Perceptual expertise and the plasticity of other-race face recognition. Vis. Cog..

[32] Kelly DJ (2009). Development of the other-race effect during infancy: Evidence toward universality?. J. Exp. Child Psychol..

[33] Turati C, Macchi Cassia V, Simion F, Leo I (2006). Newborns’ face recognition: Role of inner and outer facial features. Child Dev..

[34] Scott LS (2011). Mechanisms underlying the emergence of object representations during infancy. J. Cog. Neurosci..

[35] Kanwisher N, McDermott J, Chun MM (1997). The fusiform face area: A module in human extrastriate cortex specialized for face perception. J. Neurosci..

[36] Dehaene S (2010). How learning to read changes the cortical networks for vision and language. Sci..

[37] Friendly RH, Rendall D, Trainor LJ (2013). Plasticity after perceptual narrowing for voice perception: reinstating the ability to discriminate monkeys by their voices at 12 months of age. Frontiers in Psychol..

[38] Pascalis O (2014). On the links among face processing, language processing, and narrowing during development. Child Dev. Perspectives.

[39] Perrachione TK, Chiao JY, Wong PCM (2010). Asymmertric cultural effects in perceptual expertise underlie an own-race bias for voices. Cognition.

[40] Kinzler KD, Dupoux E, Spelke ES (2007). The native language of social cognition. Proceed. Nat. Acad. Sci..

[41] Tham Diana Su Yun, Bremner J. Gavin, Hay Dennis (2017). The other-race effect in children from a multiracial population: A cross-cultural comparison. Journal of Experimental Child Psychology.

[42] Tham DSY, Woo PJ, Bremner JG (2019). Development of the other-race effect in Malaysian-Chinese infants. Dev. Psychobiol..

[43] Crookes K, Rhodes G (2017). Poor recognition of other-race faces cannot always be explained by a lack of effort. Vis. Cog..

[44] Lane J (2018). Impacts of impaired face perception on social interactions and quality of life in age-related macular degeneration: A qualitative study and new community resources. PLoS One.

[45] Yardley L, McDermott L, Pisarski S, Duchaine BC, Nakayama K (2008). Psychosocial consequences of developmental prosopagnosia: A problem of recognition. J. Psychosomatic Res..

