# Supplementary material for: A critical period for faces: Other-race face recognition is improved by childhood but not adult social contact
Source: Sci Rep. 2019 Sep 6;9:12820. doi: 10.1038/s41598-019-49202-0 (PMC6731249; doi:10.1038/s41598-019-49202-0)
Supplement: Supplementary file 1 — McKone_face_race_critical_period_Supplementary_Info [file 41598_2019_49202_MOESM1_ESM.docx]

**SUPPLEMENTARY INFORMATION**

**A critical period for faces: Other-race face recognition is improved by childhood but not adult social contact**

Elinor McKone, Lulu Wan, Madeleine Pidcock, Kate Crookes, Katherine Reynolds,

Amy Dawel, Evan Kidd, Chiara Fiorentini

*Scientific Reports*

*Date of this version: 15th Aug 2019*

List of Supplementary Information

Tables S1 – S17

Figs S1 – S3

Appendices 1-8 (Appendix 7 includes Table S18)
References for Supplementary Information

**SUPPLEMENTARY TABLES**

***Table S1***. Range of contact scores: Rationale for participant groups, and sample sizes, included or excluded from Figure 4. Rows marked in bold had sufficient range of contact for correlations to be analysed (met criterion that the median of other-race/ethnicity classmates should be ≥10%), and are the data sets used for Figure 4 and Figure S2.

| **Age of contact** | **Participant group** | **Effect** | **Year tested** | **n** | **Median % other-group in class** | **Enough range**  **to include in**  **correlations?** |
| --- | --- | --- | --- | --- | --- | --- |
| Primary | Caucasian | ORE | 2012 | 39 | 5.0 | no |
|  | Caucasian | ORE | 2013 | 67 | 7.0 | no |
|  | **Caucasian** | **ORE** | **2015** | **57** | **10.0** | **yes** |
|  | Caucasian | ORE | 2012+2013+2015 | 163 | 9.0 | no |
|  | Asian (Eastern-raised) | ORE | 2012+2013+2015 | 105 | 0.0 | no |
|  | **Asian (Western-raised)** | **ORE** | **2012+2013+2015** | **59** | **70.0** | **yes** |
|  | **Northern-European Caucasian** | **OEE** | **2009** | **46** | **10.0** | **yes** |
| Secondary | Caucasian | ORE | 2012 | 39 | 15.0 | yes |
|  | Caucasian | ORE | 2013 | 24 | 12.5 | yes |
|  | Caucasian | ORE | 2015 | 57 | 20.0 | yes |
|  | **Caucasian** | **ORE** | **2012+2013+2015** | **120** | **16.0** | **yes** |
|  | Asian (Eastern-raised) | ORE | 2012+2013+2015 | 92 | 0.0 | no |
|  | **Asian (Western-raised)** | **ORE** | **2012+2013+2015** | **57** | **63.0** | **yes** |
|  | **Northern-European Caucasian** | **OEE** | **2009** | **46** | **20.0** | **yes** |
| Adult | Caucasian | ORE | 2012 | 39 | 28.0 | yes |
|  | Caucasian | ORE | 2013 | 24 | 29.5 | yes |
|  | Caucasian | ORE | 2015 | 57 | 35.0 | yes |
|  | **Caucasian** | **ORE** | **2012+2013+2015** | **120** | **31.5** | **yes** |
|  | **Asian (Eastern-raised)** | **ORE** | **2012+2013+2015** | **92** | **60.0** | **yes** |
|  | **Asian (Western-raised)** | **ORE** | **2012+2013+2015** | **57** | **52.0** | **yes** |
|  | **Northern-European Caucasian** | **OEE** | **2009** | **35** | **20.0** | **yes** |

***Table S2*.** **Primary School Contact:** Correlation with ORE/OEE and p- values for data in Fig 4a, plus descriptive statistics for contact variables (IV = independent variable) including skew (used to determine whether Pearson's r or nonparametric tau was most appropriate to report; if skew was significant, we analysed tau).

| **DV** | **IV (as labelled in Fig 4a)** | **Contact with people of race:** | **N for Correlation** | **Predicted direction** | **Correlation** | ***p***^[1]^ | **r or tau**^[2]^ | **Median** | **Mean** | **SD** | **Scale Range** | **Observed Range** | **Skewness** | **SE of Skewness** | **Sig. (>1.96)** | **Upper bound r**^[3]^ |
| --- | --- | --- | --- | --- | --- | --- | --- | --- | --- | --- | --- | --- | --- | --- | --- | --- |
| *Asian observers (Western-raised)* | | |  |  |  |  |  |  |  |  |  |  |  |  |  |  |
| ORE | C own | Asian | 59 | P | 0.08 | 0.39 | tau | 14 | 17.17 | 13.34 | 0-100 | 2-55 | 1.22 | 0.31 | 3.92 | 0.87 |
| ORE | F own | Asian | 59 | P | 0.20 | 0.03 | tau | 20 | 29.51 | 27.29 | 0-100 | 0-87 | 0.81 | 0.31 | 2.61 | 0.87 |
| ORE | N own | Asian | 59 | P | 0.20 | 0.03 | tau | 9 | 12.85 | 14.00 | 0-100 | 0-55 | 1.35 | 0.31 | 4.34 | 0.87 |
| ORE | H&R own | Asian | 59 | P | 0.32 | 0.01 | r | 3.3 | 3.37 | 1.03 | 1-6 | 1-5 | -0.16 | 0.31 | 0.51 | 0.83 |
| ORE | C other | Caucasian | 59 | N | -0.06 | 0.49 | tau | 70 | 69.69 | 20.66 | 0-100 | 15-96 | -1.05 | 0.31 | 3.38 | 0.87 |
| ORE | F other | Caucasian | 59 | N | -0.20 | 0.12 | r | 68 | 61.17 | 28.97 | 0-100 | 0-100 | -0.49 | 0.31 | 1.57 | 0.87 |
| ORE | N other | Caucasian | 59 | N | -0.17 | 0.07 | tau | 81 | 76.39 | 22.80 | 0-100 | 0-100 | -1.28 | 0.31 | 4.11 | 0.87 |
| ORE | H&R other | Caucasian | 59 | N | -0.22 | 0.02 | tau | 4.9 | 4.70 | 0.87 | 1-6 | 1-6 | -1.55 | 0.31 | 4.97 | 0.82 |
| *Caucasian observers* | | |  |  |  |  |  |  |  |  |  |  |  |  |  |  |
| ORE | C own | Caucasian | 57 | P | 0.29 | 0.002 | tau | 85 | 80.58 | 16.12 | 0-100 | 20-100 | -1.33 | 0.32 | 4.21 | 0.70 |
| ORE | F own | Caucasian | 57 | P | 0.23 | 0.01 | tau | 85 | 80.72 | 18.97 | 0-100 | 25-100 | -0.98 | 0.32 | 3.11 | 0.70 |
| ORE | N own | Caucasian | 57 | P | 0.27 | 0.01 | tau | 85 | 81.04 | 20.62 | 0-100 | 20-100 | -1.44 | 0.32 | 4.55 | 0.70 |
| ORE | H&R own | Caucasian | 57 | P | 0.08 | 0.40 | tau | 5.4 | 5.21 | 0.61 | 1-6 | 3.62-6 | -0.71 | 0.32 | 2.23 | 0.60 |
| ORE | C other | Asian | 57 | N | -0.33 | 0.01 | r | 10 | 11.74 | 9.15 | 0-100 | 0-31 | 0.49 | 0.32 | 1.54 | 0.70 |
| ORE | F other | Asian | 57 | N | -0.27 | 0.01 | tau | 9 | 11.32 | 12.35 | 0-100 | 0-50 | 1.14 | 0.32 | 3.60 | 0.70 |
| ORE | N other | Asian | 57 | N | -0.25 | 0.01 | tau | 8 | 10.82 | 13.29 | 0-100 | 0-70 | 2.20 | 0.32 | 6.97 | 0.70 |
| ORE | H&R other | Asian | 57 | N | -0.23 | 0.09 | r | 3.0 | 2.65 | 2.65 | 1-6 | 1-5.13 | -0.39 | 0.32 | 1.22 | 0.67 |
| *Northern-European Caucasian observers* | | |  |  |  |  |  |  |  |  |  |  |  |  |  |  |
| OEE | C own | North-Europe | 46 | P | 0.32 | 0.003 | tau | 90 | 80.24 | 20.37 | 0-100 | 10-100 | -1.83 | 0.35 | 5.22 | 0.88 |
| OEE | F own | North-Europe | 46 | P | 0.30 | 0.006 | tau | 90 | 79.78 | 22.75 | 0-100 | 0-100 | -1.74 | 0.35 | 4.98 | 0.88 |
| OEE | C other | South-Europe | 46 | N | -0.20 | 0.07 | tau | 10 | 14.03 | 13.50 | 0-100 | 0-70 | 1.96 | 0.35 | 5.60 | 0.88 |
| OEE | F other | South-Europe | 46 | N | -0.34 | 0.002 | tau | 10 | 12.57 | 15.17 | 0-100 | 0-70 | 1.93 | 0.35 | 5.52 | 0.88 |

Notes: [1] p values in red refers to p<.05, p values in blue refers to p<.09. [2] Contact demographics (IV) were skewed in many cases (see the column of “Sig. (>1.96)” in red). Thus, we report nonparametric correlations (tau) where this was the case. For the remaining cases, distributions did not differ significantly from normal, and thus Pearson's r was used. DV = dependent variable; IV = independent variable (i.e., the predictor). C = Classmates, F = Friends; N = neighbours; H&R = Hancock & Rhodes^1^ questionnaire (in childhood-contact variant^2^). [3] Upper bound r for the correlation of two variables is determined from internal reliability of each, as r _upper bound_= square root of the product of the internal reliabilities of the two tasks^3^. Internal reliability for ORE, OEE and H&R measures provided in Tables S6 & S9. For C, F, and N contact measures we have no information about reliability (because there is only one test item, i.e., the % of people); thus upper bound reliability is based on assuming a reliability of 1.0 (of course in practice it will be less, meaning the upper bound reported should be treated as overestimating the true upper bound).

***Table S3*.** **Secondary School Contact:** Correlation with ORE/OEE and p- values for data in Fig 4b, plus descriptive statistics for contact variables, including skew (which was used to determine whether Pearson's r or nonparametric tau was most appropriate to report; if skew was significant, we analysed tau).

| **DV** | **IV (as labelled in Fig 4b)** | **Contact with people of race** | **N for Correl-ation** | **Predicted direction** | | **Correlation** | ***P*** ^[1]^ | **r or tau**^[2]^ | **Median** | **Mean** | **SD** | **Scale Range** | **Observed Range** | | **Skewness** | | **SE of Skewness** | | | **Sig. of skew**  **(>1.96)** | **Upper bound r**^[3]^ |
| --- | --- | --- | --- | --- | --- | --- | --- | --- | --- | --- | --- | --- | --- | --- | --- | --- | --- | --- | --- | --- | --- |
| *Asian observers (Western-raised)* | | |  |  |  | |  |  |  |  |  |  |  |  | | | | |  | |  |
| ORE | C own | Asian | 57 | P | | -0.05 | 0.60 | tau | 27 | 26.7 | 15.6 | 0-100 | 1-80 | | 0.64 | | 0.32 | | | 2.01 | 0.87 |
| ORE | F own | Asian | 57 | P | | 0.12 | 0.37 | r | 30 | 29.2 | 19.8 | 0-100 | 0-79 | | 0.24 | | 0.32 | | | 0.77 | 0.87 |
| ORE | N own | Asian | 57 | P | | 0.11 | 0.24 | tau | 7 | 10.5 | 9.7 | 0-100 | 0-43 | | 1.49 | | 0.32 | | | 4.72 | 0.87 |
| ORE | C other | Caucasian | 57 | N | | 0.10 | 0.48 | r | 63 | 61.5 | 21.9 | 0-100 | 6-97 | | -0.58 | | 0.32 | | | 1.84 | 0.87 |
| ORE | F other | Caucasian | 57 | N | | -0.11 | 0.43 | r | 34 | 38.6 | 26.4 | 0-100 | 0-99 | | 0.53 | | 0.32 | | | 1.66 | 0.87 |
| ORE | N other | Caucasian | 57 | N | | -0.10 | 0.45 | r | 57 | 57.5 | 21.0 | 0-100 | 0-100 | | 0.16 | | 0.32 | | | 0.51 | 0.87 |
| *Caucasian observers (tested in 2012+2013+2015)* | | | | |  | |  |  |  |  |  |  |  | | |  | |  | | |  |
| ORE | C own | Caucasian | 120 | P | | 0.10 | 0.11 | tau | 70 | 69.3 | 20.0 | 0-100 | 2-100 | | -0.94 | | 0.22 | | | 4.23 | 0.82 |
| ORE | F own | Caucasian | 120 | P | | 0.04 | 0.51 | tau | 80 | 76.1 | 22.3 | 0-100 | 3-100 | | -1.17 | | 0.22 | | | 5.30 | 0.82 |
| ORE | N own | Caucasian | 120 | P | | 0.12 | 0.06 | tau | 86 | 80.6 | 19.2 | 0-100 | 24-100 | | -1.12 | | 0.22 | | | 5.31 | 0.82 |
| ORE | C other | Asian | 120 | N | | -0.06 | 0.31 | tau | 16 | 19.6 | 19.6 | 0-100 | 0-90 | | 1.64 | | 0.22 | | | 7.77 | 0.82 |
| ORE | F other | Asian | 120 | N | | -0.04 | 0.52 | tau | 11 | 15.5 | 15.5 | 0-100 | 0-95 | | 2.05 | | 0.22 | | | 9.70 | 0.82 |
| ORE | N other | Asian | 120 | N | | -0.10 | 0.14 | tau | 7 | 10.8 | 10.8 | 0-100 | 0-70 | | 1.65 | | 0.22 | | | 7.82 | 0.82 |
| *Caucasian observers (tested in 2015)* ^[4]^ | | |  |  | |  |  |  |  |  |  |  |  | |  | |  | | |  |  |
| ORE | C own | Caucasian | 57 | P | | 0.16 | 0.09 | tau | 70 | 67.3 | 20.6 | 0-100 | 5-100 | | -0.81 | | 0.32 | | | 2.57 | 0.70 |
| ORE | F own | Caucasian | 57 | P | | 0.15 | 0.12 | tau | 82 | 76.5 | 23.1 | 0-100 | 3-100 | | -1.13 | | 0.32 | | | 3.59 | 0.70 |
| ORE | N own | Caucasian | 57 | P | | 0.12 | 0.21 | tau | 81 | 78.7 | 20.9 | 0-100 | 24-100 | | -1.12 | | 0.32 | | | 3.56 | 0.70 |
| ORE | C other | Asian | 57 | N | | -0.08 | 0.41 | tau | 20 | 22.1 | 18.0 | 0-100 | 0-90 | | 1.66 | | 0.32 | | | 5.24 | 0.70 |
| ORE | F other | Asian | 57 | N | | -0.09 | 0.33 | tau | 10 | 15.7 | 18.3 | 0-100 | 0-95 | | 2.11 | | 0.32 | | | 6.68 | 0.70 |
| ORE | N other | Asian | 57 | N | | -0.07 | 0.44 | tau | 10 | 12.5 | 13.2 | 0-100 | 0-70 | | 1.74 | | 0.32 | | | 5.50 | 0.70 |
| *Northern-European Caucasian observers* | | |  |  | |  |  |  |  |  |  |  |  | |  | |  | | |  |  |
| OEE | C own | North-Europe | 46 | P | | 0.04 | 0.69 | tau | 76 | 71.7 | 19.0 | 0-100 | 20-100 | | -1.18 | | 0.35 | | | 3.38 | 0.88 |
| OEE | F own | North-Europe | 46 | P | | 0.04 | 0.70 | tau | 77 | 75.7 | 21.3 | 0-100 | 0-100 | | -1.35 | | 0.35 | | | 3.84 | 0.88 |
| OEE | C other | South-Europe | 46 | N | | 0.08 | 0.42 | tau | 20 | 22.7 | 15.9 | 0-100 | 0-73 | | 0.95 | | 0.35 | | | 2.72 | 0.88 |
| OEE | F other | South-Europe | 46 | N | | -0.11 | 0.31 | tau | 10 | 15.8 | 15.3 | 0-100 | 0-60 | | 1.17 | | 0.35 | | | 3.33 | 0.88 |

Notes: Notes [1-3] are as for Table S2.

[4] For Caucasian observers, two samples are included. N=120 is for the observers tested in 2012, 2013, and 2015. N=57 is the identical set of observers as analysed for primary contact in Table S2, showing that this n=57 groups considered by itself shows significant correlations with primary school contact (Table S2) but not secondary contact (Table S3).

***Table S4.*** **Adult Contact:** Correlation with ORE/OEE and p- values for data in Fig 4c, plus descriptive statistics for contact variables, including skew (which was used to determine whether Pearson's r or nonparametric tau was most appropriate to report; if skew was significant, we analysed tau).

| **DV** | **IV (as labelled in Fig 4c)** | **Contact with people of race:** | | **N for Correlation** | | **Predicted direction** | | **Correlation** | ***P***^[1]^ | **r or tau**^[2]^ | **Median** | | **Mean** | **SD** | **Scale Range** | **Observed Range** | **Skewness** | | **SE of Skewness** | **Sig. (>1.96)** | **Upper bound r**^[3]^ |
| --- | --- | --- | --- | --- | --- | --- | --- | --- | --- | --- | --- | --- | --- | --- | --- | --- | --- | --- | --- | --- | --- |
| *Asian observers (Western-raised)* | | | | | | | | | | | | | | | | | | | | |  |
| ORE | C own | Asian | | 57 | | P | | -0.02 | 0.80 | tau | 35 | | 34.6 | 15.0 | 0-100 | 8-91 | 0.71 | | 0.32 | 2.24 | 0.87 |
| ORE | F own | Asian | | 57 | | P | | 0.14 | 0.30 | r | 50 | | 52.2 | 30.9 | 0-100 | 0-100 | -0.06 | | 0.32 | 0.20 | 0.87 |
| ORE | N own | Asian | | 57 | | P | | 0.07 | 0.49 | tau | 15 | | 19.4 | 17.8 | 0-100 | 0-91 | 1.67 | | 0.32 | 5.28 | 0.87 |
| ORE | H&R own | Asian | | 59 | | P | | 0.12 | 0.37 | r | 4.0 | | 3.8 | 0.7 | 1-6 | 2-5 | -0.54 | | 0.31 | 1.73 | 0.80 |
| ORE | C other | Caucasian | | 57 | | N | | 0.13 | 0.35 | r | 52 | | 54.8 | 16.6 | 0-100 | 6-92 | 0.04 | | 0.32 | 0.13 | 0.87 |
| ORE | F other | Caucasian | | 57 | | N | | -0.06 | 0.64 | r | 39 | | 39.3 | 28.8 | 0-100 | 0-97 | 0.38 | | 0.32 | 1.21 | 0.87 |
| ORE | N other | Caucasian | | 57 | | N | | 0.01 | 0.90 | tau | 69 | | 69.3 | 23.1 | 0-100 | 0-100 | -0.82 | | 0.32 | 2.59 | 0.87 |
| ORE | H&R other | Caucasian | | 59 | | N | | -0.18 | 0.17 | r | 4.0 | | 4.1 | 0.9 | 1-6 | 1-6 | -0.39 | | 0.31 | 1.24 | 0.83 |
| *Caucasian observers* | |  |  | |  | |  | | |  | | | | | | | |  | | |  |
| ORE | C own | Caucasian | | 120 | | P | | 0.03 | 0.78 | r | | 60 | 57.7 | 14.8 | 0-100 | 14-100 | -0.02 | | 0.22 | 0.09 | 0.82 |
| ORE | F own | Caucasian | | 120 | | P | | 0.05 | 0.39 | tau | | 75 | 72.1 | 18.3 | 0-100 | 19-100 | -0.65 | | 0.22 | 2.92 | 0.82 |
| ORE | N own | Caucasian | | 120 | | P | | -0.01 | 0.93 | r | | 70 | 66.8 | 23.2 | 0-100 | 10-10 | -0.41 | | 0.22 | 1.84 | 0.82 |
| ORE | H&R own | Caucasian | | 163 | | P | | -.002 | 0.98 | tau | | 5.00 | 4.9 | 0.67 | 1-6 | 2.57-6 | -0.59 | | 0.19 | 3.08 | 0.76 |
| ORE | C other | Asian | | 120 | | N | | 0.03 | 0.71 | r | | 32 | 32.3 | 12.0 | 0-100 | 0-58 | -0.18 | | 0.22 | 0.82 | 0.82 |
| ORE | F other | Asian | | 120 | | N | | -0.05 | 0.41 | tau | | 14 | 18.1 | 15.6 | 0-100 | 0-81 | 1.64 | | 0.22 | 7.41 | 0.82 |
| ORE | N other | Asian | | 120 | | N | | -0.04 | 0.56 | tau | | 20 | 23.3 | 19.5 | 0-100 | 0-86 | 1.00 | | 0.22 | 4.53 | 0.82 |
| ORE | H&R other | Asian | | 163 | | N | | -0.02 | 0.83 | r | | 3.3 | 3.3 | 0.7 | 1-6 | 1.38-4.63 | -0.12 | | 0.19 | 0.64 | 0.76 |
| *Northern-European Caucasian observers* | | | | | | | | | | | | | | | | | | | | |  |
| OEE | C own | North-Europe | | 35 | | P | | 0.09 | 0.47 | tau | | 70 | 69.8 | 21.5 | 0-100 | 5-100 | -1.35 | | 0.40 | 3.38 | 0.88 |
| OEE | F own | North-Europe | | 46 | | P | | 0.05 | 0.67 | tau | | 80 | 73.5 | 21.4 | 0-100 | 2-100 | -1.24 | | 0.35 | 3.53 | 0.88 |
| OEE | C other | South-Europe | | 35 | | N | | 0.23 | 0.18 | r | | 20 | 21.0 | 13.9 | 0-100 | 0-50 | 0.26 | | 0.40 | 0.65 | 0.88 |
| OEE | F other | South-Europe | | 46 | | N | | -0.01 | 0.95 | tau | | 15 | 16.8 | 13.0 | 0-100 | 0-50 | 1.06 | | 0.35 | 3.02 | 0.88 |
| *Asian observers (Eastern-raised)* | | | | | | | | | | | | | | | | | | | | |  |
| ORE | C own | Asian | | 92 | | P | | -0.04 | 0.54 | tau | | 32 | 37.6 | 23.0 | 0-100 | 4-97 | 0.92 | | 0.25 | 3.65 | 0.74 |
| ORE | F own | Asian | | 92 | | P | | 0.10 | 0.19 | tau | | 85 | 74.9 | 25.3 | 0-100 | 3-100 | -1.16 | | 0.25 | 4.63 | 0.74 |
| ORE | N own | Asian | | 92 | | P | | 0.05 | 0.46 | tau | | 30 | 36.3 | 27.9 | 0-100 | 0-100 | 0.82 | | 0.25 | 3.28 | 0.74 |
| ORE | H&R own | Asian | | 105 | | P | | 0.07 | 0.31 | tau | | 4.1 | 4.1 | 0.8 | 1-6 | 1.38-5.25 | -1.17 | | 0.24 | 4.97 | 0.69 |
| ORE | C other | Caucasian | | 92 | | N | | 0.02 | 0.82 | tau | | 60 | 54.6 | 25.4 | 0-100 | 0-95 | -0.59 | | 0.25 | 2.37 | 0.74 |
| ORE | F other | Caucasian | | 92 | | N | | -0.11 | 0.12 | tau | | 10 | 20.4 | 22.5 | 0-100 | 0-97 | 1.55 | | 0.25 | 6.18 | 0.74 |
| ORE | N other | Caucasian | | 92 | | N | | -0.15 | 0.15 | r | | 60 | 56.5 | 29.2 | 0-100 | 0-100 | -0.46 | | 0.25 | 1.84 | 0.74 |
| ORE | H&R other | Caucasian | | 105 | | N | | -0.24 | 0.01 | r | | 3.6 | 3.6 | 1.1 | 1-6 | 1-5.86 | -0.30 | | 0.24 | 1.26 | 0.70 |
| ORE | Months in West | Caucasian | | 90 | | N | | 0.01 | 0.88 | tau | | 15 | 16.1 | 13.2 | 0+ | 1-61 | 0.99 | | 0.25 | 3.88 | 0.85 |

Notes: As for Table S3.

***Table S5*.** Sample demographics, testing details (in lab class groups or individually), and remuneration, in each sample for whom correlations are presented in Figure 4.

|  |  |  |  | **AGE** | |  | **GENDER** | | |  | | | **TESTING AND REMUNERATION** | | | | |
| --- | --- | --- | --- | --- | --- | --- | --- | --- | --- | --- | --- | --- | --- | --- | --- | --- | --- |
| **Effect tested** | **Observer sample & year/s of testing** | **Figure 4 part/s where correlation**  **appears** | **Total N** | **Mean age** | **SD age** |  | **n Male** | **n Female** | **n Other**^[1]^ | |  | **n tested in teaching lab classes (no payment)** | | **n tested individually & paid $15 for 1hr** | **n tested individually & paid $24 for 2hrs**^[2]^ | **n tested**  **individually**  **for course**  **credit** |  |
| ORE | Western-raised Asian (2012+2013+2015) | Fig 4a-c | 59 | 18.5 | 1.5 |  | 13 | 46 | 0 | |  | 14 | | 16 | 0 | 29 |  |
| ORE | Eastern-raised Asian (2012+2013+2015) ^[3]^ | Fig 4c | 92 | 22.0 | 2.4 |  | 27 | 65 | 0 | |  | 28 | | 64 | 0 | 0 |  |
| ORE | Eastern-raised Asian (2012+2013+2015) | Fig 4c | 105 | 21.8 | 2.4 |  | 32 | 73 | 0 | |  | 37 | | 68 | 0 | 0 |  |
| ORE | Caucasian  (2015) | Fig 4a | 57 | 21.1 | 4.1 |  | 15 | 42 | 0 | |  | 57 | | 0 | 0 | 0 |  |
| ORE | Caucasian (2012+2013+2015) | Fig 4b-c | 120 | 21.3 | 4.7 |  | 31 | 87 | 3 | |  | 96 | | 24 | 0 | 0 |  |
| ORE | Caucasian (2012+2013+2015) | Fig 4c | 163 | 21.1 | 4.2 |  | 42 | 118 | 3 | |  | 135 | | 28 | 0 | 0 |  |
| OEE | North-European Caucasian (2009) | Fig 4a-c | 46 | 22.2 | 2.8 |  | 15 | 31 | N/A | |  | 0 | | 0 | 46 | 0 |  |

Notes:

[1] From 2012, our ethics board has required us to allow an "Other" option for gender.

[2] For the OEE participants, the 2 hrs of testing included some additional tests not relevant here (e.g., CFMT-Australian 24hr delay condition) and for which data are reported in McKone et al^4^.

[3] Eastern-raised Asians appear in two rows to allow us to present demographics separately for the sample that had H&R adult scores in Fig 4c (n=105), and the subsample with C/F/N scores (n=92). Caucasians appear in three rows to allow us to present demographics separately for sample (2015 only, n=57) used for primary school contact in Fig 4, and the larger samples used for secondary and adult contact (2012+2013+2015) in Fig 4 (n=163 for adult H&R, n=120 for other contact measures). Note the sample analysed for primary school contact effects is very similar in mean age (approx 21 yrs) and gender distribution (approx 73% female) to the samples used to evaluate secondary and adult correlations.

***Table S6.*** Reliability and measurement error for CFMT tasks and ORE scores: In each sample for whom correlations are presented in Fig 4, table lists internal reliability (Cronbach's alpha) of each CFMT test, reliability of the ORE or OEE difference scores, and resulting measurement error (68%CI) in an individual participant's ORE or OEE score.

| **Observer sample & year/s of testing** | **Figure 4 part/s where correlation**  **appears** | **N** | **Alpha (Own-race/**  **ethnicity CFMT)** | **Alpha**  **(Other-race/**  **ethnicity CFMT)** | **Correlation between CFMT-own and CFMT-other** ^[2]^ **(r)** | **Reliability of the ORE (or OEE) scores** ^[3]^ | **Mean ORE**  **(or OEE)** | **SD ORE**  **(or OEE)** | **68%CI on individual's ORE (or OEE) score** ^[4]^ |
| --- | --- | --- | --- | --- | --- | --- | --- | --- | --- |
| Western-raised Asian (2012+2013+2015) | Fig 4a-c | 59 | 0.89 | 0.85 | 0.603 | 0.671 | 1.01 | 9.75 | ±5.6 |
| Eastern-raised Asian (2012+2013+2015) | Fig 4c | 92 | 0.89 | 0.81 | 0.669 | 0.554 | 8.60 | 9.44 | ±6.3 |
| Eastern-raised Asian (2012+2013+2015) ^[1]^ | Fig 4c | 105 | 0.89 | 0.81 | 0.668 | 0.544 | 8.51 | 9.31 | ±6.3 |
| Caucasian (2015) | Fig 4a | 57 | 0.81 | 0.85 | 0.673 | 0.486 | 6.58 | 8.83 | ±6.3 |
| Caucasian (2012+2013+2015) | Fig 4b-c | 120 | 0.83 | 0.85 | 0.691 | 0.469 | 5.98 | 8.57 | ±6.2 |
| Caucasian (2012+2013+2015) | Fig 4c | 163 | 0.83 | 0.85 | 0.640 | 0.556 | 7.25 | 9.38 | ±6.3 |
| Norther-European Caucasian (2009) | Fig 4a-c | 46 | 0.88 | 0.88 | 0.646 | 0.667 | 5.62 | 9.00 | ±5.2 |

Notes:

[1] For explanation of the various sample sizes for Eastern-raised Asians and for Caucasians, see Table S1 and Appendix 7.

[2] These correlations are needed to calculate the reliability of the ORE/OEE difference score (i.e., the own-minus-other scores).

[3] Formula for reliability of the ORE/OEE scores is the standard method of calculating the reliability of a difference score^5^, i.e., Reliability of difference scores = {[(r_xx_ + r_yy_)/2] - r_xy_}/ (1 - r_xy_), Where r_xx_ = reliability of condition x; r_yy_ = reliability of condition y; r_xy_ = correlation between condition x and condition y.

[4] The measurement error (95%CI) on an individual participant's ORE (or OEE) score is calculated using formula^6^: 68% CI on an individual's test score = score ± sd of sample * sqrt (1-alpha). To convert 68%CI to 95%CI, multiply by 1.96.

***Table S7*.** Marriage attitude: Descriptive statistics for the marriage attitude variable, for the samples used in correlations in Figure S2.

| **Observer**  **Sample** | **Figure S2 part/s where correlation appears** | **N** | **Explanation of N** | **Median** | **Mean** | **SD** | **Scale Range** | **Observed Range** | **Skewness** | **SE of Skewness** | **Sig. (>1.96 or <-1.96)** |
| --- | --- | --- | --- | --- | --- | --- | --- | --- | --- | --- | --- |
| Western-raised Asian | Fig S2A | 59 | For primary C/F/N contact | 4.0 | 4.3 | 2.1 | 1-9 | 1-9 | 0.45 | 0.31 | 1.46 |
| Western-raised Asian | Fig S2B-C | 57 | For sec and adult C/F/N contact | 4.0 | 4.4 | 2.1 | 1-9 | 1-9 | 0.41 | 0.32 | 1.31 |
| Western-raised Asian | Fig S2A-C | 59 | For H&R contact | 4.0 | 4.3 | 2.1 | 1-9 | 1-9 | 0.45 | 0.31 | 1.46 |
| Eastern-raised Asian | Fig S2C | 92 | For adult C/F/N contact | 4.0 | 3.6 | 1.7 | 1-9 | 1-8 | 0.33 | 0.25 | 1.33 |
| Eastern-raised Asian | Fig S2C | 105 | For H&R contact | 4.0 | 3.5 | 1.7 | 1-9 | 1-8 | 0.40 | 0.24 | 1.71 |
| Caucasian | Fig S2A-C | 57 | For primary C/F/N contact | 4.0 | 4.1 | 1.6 | 1-9 | 2-8 | 0.81 | 0.32 | 2.56 |
| Caucasian | Fig S2B-C | 120 | For sec and adult C/F/N contact | 4.0 | 3.9 | 1.4 | 1-9 | 1-8 | 0.64 | 0.22 | 2.90 |
| Caucasian | Fig S2A-C | 163 | For H&R contact | 4.0 | 3.8 | 1.3 | 1-9 | 1-8 | 0.62 | 0.19 | 3.24 |

Note:

Values in red indicate samples in which marriage attitude was significantly skewed. In Fig S2, we report tau as the correlation measures if either variable (marriage attitude, or contact predictor) has significant skew; otherwise Pearson's r is reported.

***Table S8***. Other-race/ethnicity effect scores: Descriptive statistics for the ORE or OEE variable, for the samples used in correlations in Figure 4, plus significance test results comparing the mean ORE/OEE to zero.

| **Variable** | **Observer Sample** | **Figure 4 part/s where correlation appears** | **N** | **Explanation of N** | **Median**  **(own-other in % correct)** | **Mean**  **(own-other in % correct)** | **SD** | **Sig ORE/**  **OEE** | **Scale Range** | **Observed Range** | **Skewness** | **SE of Skewness** | **Sig. (>1.96 or <-1.96)** ^[1]^ |
| --- | --- | --- | --- | --- | --- | --- | --- | --- | --- | --- | --- | --- | --- |
| ORE^[2]^ | Western-raised Asian | Fig 4a | 59 | For primary C/F/N contact | 2.0 | 1.0 | 9.7 | p=.429 | -100-100 | -27-18 | 0.49 | 0.31 | 1.56 |
| ORE | Western-raised Asian | Fig 4b-c | 57 | For sec and adult C/F/N contact | 2.0 | 0.8 | 9.6 | p=.431 | -100-100 | -27-18 | 0.55 | 0.32 | 1.73 |
| ORE | Western-raised Asian | Fig 4a-c | 59 | For H&R contact | 2.0 | 1.0 | 9.7 | p=.429 | -100-100 | -27-18 | 0.49 | 0.31 | 1.56 |
| ORE | Eastern-raised Asian | Fig 4c | 92 | For adult C/F/N contact | 10.0 | 8.6 | 9.4 | p<.001 | -100-100 | -16.67-29 | -0.29 | 0.25 | -1.16 |
| ORE | Eastern-raised Asian | Fig 4c | 105 | For H&R contact | 9.0 | 8.5 | 9.3 | p<.001 | -100-100 | -16.67-29 | -0.30 | 0.24 | -1.28 |
| ORE | Caucasian | Fig 4a-c | 57 | For primary C/F/N contact | 6.9 | 6.6 | 8.8 | p<.001 | -100-100 | -11.11-22.22 | -0.14 | 0.32 | -0.45 |
| ORE | Caucasian | Fig 4b-c | 120 | For sec and adult C/F/N contact | 6.4 | 6.0 | 8.6 | p<.001 | -100-100 | -11.11-26 | 0.10 | 0.22 | 0.43 |
| ORE | Caucasian | Fig 4a-c | 163 | For H&R contact | 6.9 | 7.2 | 9.4 | p<.001 | -100-100 | -15-41 | 0.32 | 0.19 | 1.66 |
| OEE | North-European Caucasian | Fig 4a-c | 46 | For contact across age | 4.2 | 5.6 | 9.0 | p<.001 | -100-100 | -16.66-25 | 0.28 | 0.35 | 0.80 |

Notes.

[1] Note that the ORE and OEE scores did not show significant skew for any samples; it is for this reason that the choice of whether to use r or tau in Fig 4 was determined by whether the *contact* variable (i.e., the other variable in the correlation) was significantly skewed.

[2] ORE and OEE scores are calculated as "% correct for own minus % correct for other".

***Table S9***. Internal reliability of the H&R contact scales.

| **Observer sample & year/s of testing** | **Figure 4 part/s where correlation appears** | **N** | **Own-race childhood contact H&R (Cronbach's Alpha)** | **Other-race childhood contact H&R (Cronbach's Alpha)** | **Own-race adult contact H&R (Cronbach's Alpha)** | **Other-race-race adult contact H&R (Cronbach's Alpha)** |  |
| --- | --- | --- | --- | --- | --- | --- | --- |
| Western-raised Asian (2012+2013+2015) | | Fig 4a-c | 59 | 0.894 | 0.881 | 0.831 | 0.907 |
| Eastern-raised Asian (2012+2013+2015) ^[1]^ | | Fig 4c | 105 | 0.930 | 0.910 | 0.865 | 0.905 |
| Caucasian (2015) | | Fig 4a | 57 | 0.741 | 0.910 | 0.795 | 0.850 |
| Caucasian (2012+2013+2015) | | Fig 4c | 163 | 0.816 | 0.905 | 0.827 | 0.834 |

Notes:

[1] For explanation of the various sample sizes for Eastern-raised Asians and for Caucasians, see Table S1 and Appendix 7.

***Table S10*.** **Raw Memory-for-Other-Race scores: correlation with** **Primary School Contact.** Correlation of other-race/ethnicity CFMT memory score with primary school other-race/ethnicity contact, with p-value plus measure reported (Pearson's r, or nonparametric tau if skew in contact variable was significant).

| **DV**  **(CFMT-Other)** | **IV**  **(amount of other-race/ethnicity**  **contact)** | **Contact with people of race:** | | **N for Correlation** | | | **Predicted direction** ^[1]^ | | **Correlation** | | ***p***^[2]^ | | **r or tau** |
| --- | --- | --- | --- | --- | --- | --- | --- | --- | --- | --- | --- | --- | --- |
| *Asian observers (Western-raised)* | | |  | |  |  | |  | |  | |  |  |
| CFMT-Australian | C other | Caucasian | | | 59 | | P | | -0.06 | | 0.54 | | tau |
| CFMT-Australian | F other | Caucasian | | | 59 | | P | | -0.15 | | 0.26 | | r |
| CFMT-Australian | N other | Caucasian | | | 59 | | P | | 0.05 | | 0.58 | | tau |
| CFMT-Australian | H&R other | Caucasian | | | 59 | | P | | 0.09 | | 0.33 | | tau |
| *Caucasian observers* | | |  | |  |  | |  | |  | |  |  |
| CFMT-Chinese | C other | Asian | | | 57 | | P | | 0.22 | | 0.10 | | r |
| CFMT-Chinese | F other | Asian | | | 57 | | P | | 0.11 | | 0.27 | | tau |
| CFMT-Chinese | N other | Asian | | | 57 | | P | | 0.13 | | 0.18 | | tau |
| CFMT-Chinese | H&R other | Asian | | | 57 | | P | | 0.13 | | 0.35 | | r |
| *Northern-European Caucasian observers* | | |  | |  |  | |  | |  | |  |  |
| CFMT-Original | C other | South-Europe | | | 46 | | P | | 0.24 | | 0.03 | | tau |
| CFMT-Original | F other | South-Europe | | | 46 | | P | | 0.15 | | 0.17 | | tau |

Notes: [1] Prediction direction always positive because, if contact matters at a given age, greater other-race contact should result in better other-race memory.

[2] p values in red refers to p<.05, p values in blue refers to p<.10.

***Table S11*.** **Raw Memory-for-Other-Race scores: correlation with** **Secondary School Contact.** Correlation of other-race/ethnicity CFMT memory score with secondary school other-race/ethnicity contact, with p-value plus measure reported (Pearson's r, or nonparametric tau if skew in contact variable was significant).

| **DV**  **(CFMT-Other)** | **IV**  **(amount of other-race/ethnicity**  **contact)** | **Contact with people of race:** | | **N for Correlation** | | | **Predicted direction** | | **Correlation** | | ***p*** | | **r or tau** |
| --- | --- | --- | --- | --- | --- | --- | --- | --- | --- | --- | --- | --- | --- |
| *Asian observers (Western-raised)* | | |  | |  |  | |  | |  | |  |  |
| CFMT-Australian | C other | Caucasian | | | 57 | | P | | -0.00 | | 0.99 | | r |
| CFMT-Australian | F other | Caucasian | | | 57 | | P | | 0.12 | | 0.38 | | r |
| CFMT-Australian | N other | Caucasian | | | 57 | | P | | 0.06 | | 0.66 | | r |
| *Caucasian observers* | | |  | |  |  | |  | |  | |  |  |
| CFMT-Chinese | C other | Asian | | | 120 | | P | | 0.03 | | 0.63 | | tau |
| CFMT-Chinese | F other | Asian | | | 120 | | P | | -0.05 | | 0.41 | | tau |
| CFMT-Chinese | N other | Asian | | | 120 | | P | | 0.03 | | 0.64 | | tau |
| *Northern-European Caucasian observers* | | |  | |  |  | |  | |  | |  |  |
| CFMT-Original | C other | South-Europe | | | 46 | | P | | 0.05 | | 0.62 | | tau |
| CFMT-Original | F other | South-Europe | | | 46 | | P | | 0.13 | | 0.22 | | tau |

***Table S12.*** **Raw Memory-for-Other-Race scores: correlation with** **Adult Contact.** Correlation of other-race/ethnicity CFMT memory score with adult other-race/ethnicity contact, with p-value plus measure reported (Pearson's r, or nonparametric tau if skew in contact variable was significant).

| **DV**  **(CFMT-Other)** | **IV**  **(amount of other-race/ethnicity**  **contact)** | **Contact with people of race:** | | **N for Correlation** | | | **Predicted direction** | | **Correlation** | | ***p*** | | **r or tau** |
| --- | --- | --- | --- | --- | --- | --- | --- | --- | --- | --- | --- | --- | --- |
| *Asian observers (Western-raised)* | | |  | |  |  | |  | |  | |  |  |
| CFMT-Australian | C other | Caucasian | | | 57 | | P | | 0.04 | | 0.76 | | r |
| CFMT-Australian | F other | Caucasian | | | 57 | | P | | 0.15 | | 0.26 | | r |
| CFMT-Australian | N other | Caucasian | | | 57 | | P | | 0.06 | | 0.49 | | tau |
| CFMT-Australian | H&R other | Caucasian | | | 59 | | P | | 0.12 | | 0.36 | | r |
| *Caucasian observers* | | |  | |  |  | |  | |  | |  |  |
| CFMT-Chinese | C other | Asian | | | 120 | | P | | -0.11 | | 0.25 | | r |
| CFMT-Chinese | F other | Asian | | | 120 | | P | | -0.6 | | 0.36 | | tau |
| CFMT-Chinese | N other | Asian | | | 120 | | P | | 0.02 | | 0.80 | | tau |
| CFMT-Chinese | H&R other | Asian | | | 163 | | P | | -0.10 | | 0.21 | | r |
| *Northern-European Caucasian observers* | | |  | |  |  | |  | |  | |  |  |
| CFMT-Original | C other | South-Europe | | | 35 | | P | | 0.10 | | 0.59 | | r |
| CFMT-Original | F other | South-Europe | | | 46 | | P | | -0.07 | | 0.50 | | tau |
| *Asian observers (Eastern-raised)* | | |  | |  |  | |  | |  | |  |  |
| CFMT-Australian | C other | Caucasian | | | 92 | | P | | -0.01 | | 0.86 | | tau |
| CFMT-Australian | F other | Caucasian | | | 92 | | P | | -0.01 | | 0.92 | | tau |
| CFMT-Australian | N other | Caucasian | | | 92 | | P | | 0.44 | | 0.68 | | r |
| CFMT-Australian | H&R other | Caucasian | | | 105 | | P | | -0.01 | | 0.93 | | r |
| CFMT-Australian | Time-in-West | Caucasian | | | 90 | | P | | -0.05 | | 0.51 | | tau |

***Table S13*.** Contact correlations across all combinations of the three life stages.

| **Sample** | **Contact measure** | **Primary with Secondary** | **Primary with Adult** | **Secondary with Adult** |
| --- | --- | --- | --- | --- |
| Western-raised Asians for ORE (n=57/59) ^[1]^ | Class | .48*^[2]^ | .26 | .34* (r)^[3]^ |
|  | Friends | .46* (r) | .44* (r) | .72* (r) |
|  | Neighbours | .59* | .41* | .38* |
|  | H&R | N/A | .19* | N/A |
| Caucasian for ORE (n=57/120) ^[4]^ | Class | .33* | -.04 | .13 |
|  | Friends | .37* | .22* | .39* |
|  | Neighbours | .58* | .03 | .17* |
|  | H&R | N/A | .25* | N/A |
| Eastern-raised Asian for ORE | all | N/A ^[5]^ | N/A | N/A |
| North-European Caucasian for OEE (n=46) | Class | .47* | .21 | .55* |
|  | Friends | .58* | .38* | .49* |

Notes:

[1] For Western-raised Asians, N=59 for correlations of H&R childhood with H&R adult contact; N=57 for all other correlations; N/A = correlation cannot be calculated because includes secondary as one of the variables, and H&R not measured for secondary.

[2] * = correlation is significant (p<.05).

[3] Correlations are tau except where marked as r.

[4] For Caucasians, N=57 applies to the correlation between primary and secondary school, and between primary school and adult; N=120 applies for the correlation between secondary school and adult; N/A = correlation cannot be calculated because includes secondary as one of the variables, and H&R not measured for secondary.

[5] For Eastern-raised Asians, all correlations N/A because range of contact in primary and secondary school is too low to allow valid correlations to be calculated.

***Table S14.*** **Intercorrelations between contact measures: Primary School Contact.** Results confirm the theoretical expectation that different contact-type measures (C, F, N, HR) tap different constructs (i.e., generally only modestly intercorrelated), and also that own-race and other-race contact measures (e.g., C-own and C-other) are not simply redundant (many correlations in red well below 1) as expected given the presence of third-party race/ethnicities (e.g., Indian, African, Indigenous Australian) other than the target groups participants were asked about in the contact questionnaires.

**A. Asian observers Western raised (tested for ORE)**

|  | **C-own** | **F-own** | **N-own** | **HR-own** | **C-other** | **F-other** | **N-other** | **HR-other** |
| --- | --- | --- | --- | --- | --- | --- | --- | --- |
| **C-own** | – | 0.50 | 0.53 | 0.42 | -0.67 | -0.52 | -0.49 | -0.27 |
| **F-own** |  | **–** | 0.49 | 0.53 | -0.41 | -0.76 | -0.36 | -0.40 |
| **N-own** |  |  | – | 0.35 | -0.48 | -0.44 | -0.69 | -0.25 |
| **HR-own** |  |  |  | – | -0.41 | -0.67 | -0.32 | -0.33 |
| **C-other** |  |  |  |  | – | 0.56 | 0.62 | 0.28 |
| **F-other** |  |  |  |  |  | – | 0.47 | 0.38 |
| **N-other** |  |  |  |  |  |  | – | 0.27 |

**B. Caucasian observers (tested for ORE) C. Northern European observers (tested for OEE)**

|  | **C-own** | **F-own** | **N-own** | **HR-own** | **C-other** | **F-other** | **N-other** | **HR-other** |  |  | **C-own** | **F-own** | **C-other** | **F-other** |
| --- | --- | --- | --- | --- | --- | --- | --- | --- | --- | --- | --- | --- | --- | --- |
| **C-own** | – | 0.55 | 0.46 | 0.40 | -0.72 | -0.50 | -0.37 | -0.44 |  | **C-own** | – | 0.53 | -0.71 | -0.49 |
| **F-own** |  | – | 0.35 | 0.44 | -0.40 | -0.74 | -0.28 | -0.32 |  | **F-own** |  | – | -0.36 | -0.65 |
| **N-own** |  |  | – | 0.28 | -0.37 | -0.33 | -0.82 | -0.30 |  | **C-other** |  |  | – | 0.52 |
| **HR-own** |  |  |  | – | -0.28 | -0.38 | -0.22 | -0.36 |  |  |  |  |  |  |
| **C-other** |  |  |  |  | – | 0.57 | 0.40 | 0.65 |  |  |  |  |  |  |
| **F-other** |  |  |  |  |  | – | 0.35 | 0.44 |  |  |  |  |  |  |
| **N-other** |  |  |  |  |  |  | – | 0.35 |  |  |  |  |  |  |

Notes: Correlation values are Pearson's r only if both contact measures did not show significant skew (Table S2); all other correlations are tau

***Table S15.*** **Intercorrelations between contact measures: Secondary School Contact.** Results are similar to description for primary school contact (Table S15).

**A. Asian observers Western raised (tested for ORE)**

|  | **C-own** | **F-own** | **N-own** | **C-other** | **F-other** | **N-other** |
| --- | --- | --- | --- | --- | --- | --- |
| **C-own** | – | 0.36 | 0.37 | -0.76 | -0.40 | -0.39 |
| **F-own** |  | **–** | 0.12 | -0.26 | -0.81 | -0.15 |
| **N-own** |  |  | – | -0.38 | -0.18 | -0.55 |
| **C-other** |  |  |  | – | 0.48 | 0.54 |
| **F-other** |  |  |  |  | – | 0.6 |

**B. Caucasian observers (tested for ORE) C. Northern European observers (tested for OEE)**

|  | **C-own** | **F-own** | **N-own** | **C-other** | **F-other** | **N-other** |  |  | **C-own** | **F-own** | **C-other** | **F-other** |
| --- | --- | --- | --- | --- | --- | --- | --- | --- | --- | --- | --- | --- |
| **C-own** | – | 0.52 | 0.38 | -0.73 | -0.45 | -0.39 |  | **C-own** | – | 0.49 | -0.70 | -0.40 |
| **F-own** |  | – | 0.29 | -0.41 | -0.72 | -0.28 |  | **F-own** |  | – | -0.36 | -0.48 |
| **N-own** |  |  | – | -0.27 | -0.22 | -0.77 |  | **C-other** |  |  | – | 0.41 |
| **C-other** |  |  |  | – | 0.46 | 0.36 |  |  |  |  |  |  |
| **F-other** |  |  |  |  | – | 0.28 |  |  |  |  |  |  |

Notes: Correlation values are Pearson's r only if both contact measures did not show significant skew (Table S3); all other correlations are tau

***Table S16.*** **Intercorrelations between contact measures: Adult Contact.** Results are similar to description for primary school contact (Table S15).

**A. Asian observers Western raised (tested for ORE)**

|  | **C-own** | **F-own** | **N-own** | **HR-own** | **C-other** | **F-other** | **N-other** | **HR-other** |
| --- | --- | --- | --- | --- | --- | --- | --- | --- |
| **C-own** | – | 0.38 | 0.22 | 0.23 | -0.67 | -0.40 | -0.15 | -0.27 |
| **F-own** |  | **–** | 0.12 | 0.75 | -0.25 | -0.94 | -0.12 | -0.78 |
| **N-own** |  |  | – | 0.13 | -0.25 | -0.17 | -0.71 | -0.05 |
| **HR-own** |  |  |  | – | -0.18 | -0.74 | -0.03 | -0.59 |
| **C-other** |  |  |  |  | – | 0.39 | 0.33 | 0.30 |
| **F-other** |  |  |  |  |  | – | 0.14 | 0.76 |
| **N-other** |  |  |  |  |  |  | – | 0.03 |

**B. Caucasian observers (tested for ORE) C. Northern European observers (tested for OEE)**

|  | **C-own** | **F-own** | **N-own** | **HR-own** | **C-other** | **F-other** | **N-other** | **HR-other** |  |  | **C-own** | **F-own** | **C-other** | **F-other** |
| --- | --- | --- | --- | --- | --- | --- | --- | --- | --- | --- | --- | --- | --- | --- |
| **C-own** | – | 0.18 | 0.34 | 0.04 | -0.84 | -0.16 | -0.20 | -0.19 |  | **C-own** | – | 0.18 | -0.84 | -0.16 |
| **F-own** |  | – | 0.26 | 0.39 | -0.04 | -0.71 | 0.21 | -0.40 |  | **F-own** |  | – | -0.04 | -0.71 |
| **N-own** |  |  | – | 0.15 | -0.15 | -0.17 | -0.80 | -0.28 |  | **C-other** |  |  | – | 0.07 |
| **HR-own** |  |  |  | – | 0.01 | -0.38 | -0.06 | -0.16 |  |  |  |  |  |  |
| **C-other** |  |  |  |  | – | 0.07 | 0.14 | 0.10 |  |  |  |  |  |  |
| **F-other** |  |  |  |  |  | – | 0.19 | 0.47 |  |  |  |  |  |  |
| **N-other** |  |  |  |  |  |  | – | 0.18 |  |  |  |  |  |  |

**D. Asian observers Eastern raised (tested for ORE)**

|  | **C-own** | **F-own** | **N-own** | **HR-own** | **C-other** | **F-other** | **N-other** | **HR-other** | **Time-in-West** |
| --- | --- | --- | --- | --- | --- | --- | --- | --- | --- |
| **C-own** | – | 0.23 | 0.20 | 0.19 | -0.81 | -0.26 | -0.22 | -0.14 | -0.22 |
| **F-own** |  | **–** | 0.29 | 0.32 | -0.24 | -0.88 | -0.28 | -0.47 | -0.20 |
| **N-own** |  |  | – | 0.26 | -0.17 | -0.28 | -0.82 | -0.21 | -0.05 |
| **HR-own** |  |  |  | – | -0.19 | -0.30 | -0.25 | -0.34 | -0.16 |
| **C-other** |  |  |  |  | – | 0.27 | 0.27 | 0.17 | 0.21 |
| **F-other** |  |  |  |  |  | – | 0.30 | 0.44 | 0.12 |
| **N-other** |  |  |  |  |  |  | – | 0.40 | 0.08 |
| **HR-other** |  |  |  |  |  |  |  | – | 0.17 |

Notes: Correlation values are Pearson's r only if both contact measures did not show significant skew (Table S4); all other correlations are tau

**Table S17**. Simulation outcomes for age-of-contact conditions from 1 million runs. Rows in bold (total evidence pattern) are the values reported in the article.

| **Evidence pattern** | **Number**  **of runs producing this pattern** | **Probability of**  **Type I error** |
| --- | --- | --- |
| **PRIMARY Total Evidence**: All 20 of the 20 in predicted direction, with ≥16 at p<.09,  and ≥13 significant at p<.05 | **0** | ***p*<.000001** |
| **SECONDARY Total Evidence**: 13 of the 16 in predicted direction, with ≥1 p<.09 | **63,496** | ***p*=.063496** |
| **ADULT Total Evidence**: 19 of the 29 in predicted direction, with ≥1 p<.05 | **119,331** | ***p*=.119331** |
| PRIMARY Less-strict outcome ^[1]^: ≥13 of the 20 significant at p<.05 in predicted direction | 2 | *p*=.000002 |
| PRIMARY Less-strict outcome: All 20 in predicted direction | 4,691 | *p*=.004691 |
| SECONDARY Less-strict outcome: 13 of the 16 in predicted direction ^[2]^ | 87,765 | *p*=.087765 |
| ADULT Less-strict outcome: 19 of the 29 in predicted direction | 189,695 | *p*=.189695 |

Notes:

[1] We include probabilities for various less strict definitions of the evidence pattern we obtained, for readers' interest.

[2] Probability of getting 13/16 in correct direction for our data set (p=.0878) is different from the probability of simply getting 13 heads out of 16 on a head-tails coin flip (where *p*=.0085); this is because the coin flip situation assumes all measures are independent, which is not the case here (i.e., because the different contact measures are intercorrelated).

***Figure S1***. Scatterplot of ORE against Time in West for Eastern-raised Asian observers (n=90).

**Figure S1.** ORE does not decrease with increasing amount of time Eastern-raised Asians have lived in the West (months since arriving in Australia). This is despite a wide range of durations ranging from 1 month to 61 months (i.e., just over 5 years). Note: Our results for Time in West might appear at first glance to conflict with those of Hancock and Rhodes^1^ who found a significant correlation with a variable of the same name. However, our participants all arrived in Australia as adults, while the ages reported for Hancock and Rhodes' participants indicates that the group includes Asians who arrived in Australia well before adulthood; this implies their Time in West measure was almost certainly confounded with whether contact began in childhood or adulthood.

***Figure S2.* Adult and secondary contact measures are not unreliable.**

**Figure S2. Adult and secondary contact measures are not unreliable.** If unreliability of contact scores explained lack of adult and secondary correlations with ORE/OEE (in Figure 4), then the contact scores would not correlate meaningfully with any other measures either; in contrast, results show many predicted-direction significant correlations with preference for marrying an own- versus other-race person (see Method for marriage attitude question). Predicted direction based on previous findings^7-9^.

***Figure S3.* Evidence the ORE in our cultural setting is not due to negative social attitudes.**

**Figure S3**. **Evidence the ORE in our cultural setting is not due to negative social attitudes. A.** OREs caused by negative social attitudes predict a negative correlation with willingness to marry an other-race person, i.e., larger ORE associated with with decreased willingness to marry an other-race person. For two of our three ORE samples, data reject this prediction. **B.** For the one group that showed a significant bivariate correlation (Western-raised Asians), model shown indicates how the ORE could be correlated with negative social attitudes in the Western-raised Asian group (but not the other groups), even if the ORE is caused by perceptual not social factors, in terms of an indirect, non-causal relationship. Note that testing statistically for complex patterns of relationships as suggested in this figure was not possible with our data, due to child highly non-normal child contact distributions invalidating multiple regression. For evidence that correlation between adult and child contact was stronger in our Western-raised Asian sample than our other two samples, see Table 1.

**SUPPLEMENTARY TEXT**

**Appendix 1 Explanation of participants overlapping with other studies from our lab.**

As noted in the Method, we obtained the largest sample sizes we could by collating data across several studies conducted partially for other purposes. Here, we specify how many participants overlap with other papers from our lab. Note that in all cases, their CFMT data in those other papers was analysed in a different manner to address different questions (e.g., to evaluate motivation-to-individuate effects on mean ORE in our cultural setting; to provide norms for prosopagnosia diagnosis in Asian observers on the CFMT-Chinese). None of the previous papers used the C, F, N contact questionnaire reported here. Also, no previous analysis included correlational analysis of ORE/OEE scores with any contact measures at different life stages, the critical question addressed here.

• For the Eastern-raised Asian participants analysed here for the ORE:

• n=86 had originally been tested for the studies reported in Wan et al.^2^ — all from the standard instructions condition of that paper, i.e., without specific "motivation to individuate" instructions) — and were also reported in Study 1 of Wan et al.^10^.

• An additional n=19 tested in 2015 have not been reported in any previous articles.

• For the Caucasian participants analysed here for the ORE:

• n=106 had also originally been tested for Wan et al.^2^ — all from the standard instructions condition of that paper, i.e., without specific "motivation to individuate" instructions) — and were also reported in Study 1 of Wan et al.^10^.

• An additional n=57 tested in 2015 have not been reported in any previous articles.

• For the Western-raised Asian participants analysed here for the ORE:

• n=52 had originally been tested for the studies reported in Wan et al.^2^ — all from the standard instructions condition of that paper, i.e., without specific "motivation to individuate" instructions) — and were also reported in Study 2 of Wan et al.^10^.

• An additional n=7 tested in 2015 have not been reported in any previous articles.

• For Northern-European Caucasian participants analysed here for the OEE:

• The sample of n=46 had originally been tested as part of the development and norming of the CFMT-Australian^4^, and their group-mean own-ethnicity and other-ethnicity scores were also analysed in McKone et al.^11^.

**Appendix 2: The Cambridge Face Memory Test (CFMT) tasks**

***The standard procedure for CFMT tasks:***

In Stage 1 (same images, 18 trials), participants are required to learn 3 images of one individual in different views (a left 1/3 profile, a frontal view, and a right 1/3 profile), to encourage face rather than photograph learning. Each learn image is presented sequentially, for 3 s per image. The next trial then presents three test images simultaneously in 1/3 profile and the participant has to choose which individual was just displayed; the target face is shown in the same image (i.e., same photograph) as on the learn trial. This is repeated for frontal and 2/3 profile test trials. The whole learn-and-test procedure is then repeated, for 6 to-be-learned target individuals in total (18 test trials). At the end of Stage 1, a reminder slide showing all 6 targets in frontal view appears for 20 s.

In Stage 2 (novel images, 30 trials), learned targets are now tested in new images (i.e., different lighting and/or viewpoint from Stage 1). Each of 30 test trial presents three images — a novel image of one of the 6 target individuals plus two distractor people — and participants must choose the learned target.

Stage 3 (novel images plus noise, 24 trials) is similar to Stage 2 except that now visual noise is added to the images to make the task more difficult. The lighting and/or viewpoint of the target faces is different from that in both Stage 1 and Stage 2.

Scoring: We calculated accuracy scores as percentage correct for all 72 trials.

***Face stimuli:***

Hair and clothing are excluded from all the images, so that successful performance must rely on memory of facial information. Face images averaged 5.2° vertical visual angle (5.5 cm tall viewed at 60 cm) (Caucasian, Western-raised Asian and Eastern-raised Asian Samples) and 5.4° vertical visual angle (5.2 cm tall viewed at 55 cm) (Northern-European Caucasian Sample).

For the CFMT-Chinese, all faces were Han Chinese students photographed in Beijing^11^. Concerning the ethnicity difference between the two Caucasian-face tests (CFMT-Australian and CFMT-original), the following is quoted from McKone et al^11^ (*p 4*).

"The CFMT-original faces were photographed at Harvard University, drawing on Harvard students and the Boston community. We contrasted this test with the CFMT-Australian, for which faces were photographed in Canberra at the Australian National University. The Harvard/Boston and Canberra populations differ in demographics. In McKone et al^4^, we estimated the proportion of various demographic groups to be: Jewish 35% (Harvard) versus less than 0.5% (Canberra, Australia); Italian 11.8% (Boston) versus 2.6% (Canberra); and, in contrast, British 71% (Canberra) versus 33% (Boston). Correspondingly, there are physiognomic differences between the ‘‘average face’’ created by morphing together the CFMT-original face stimuli, and the average face for the CFMT-Australian [see present article Figure 2]. These can be described as the Harvard average being somewhat more Southern European or Mediterranean in appearance and the Australian average more British or Northern European in appearance. (Note that this does not, of course, mean that all faces in the stimulus sets differ in ethnicity — there are some CFMT-original faces that appear British, and some CFMT-Australian faces that appear Southern European — but it indicates that, on average there are ethnicity differences between the sets)."

***Reason for choosing CFMT tasks as the preferred task type*.**

For a correlational design, as used in our article, accurate measurement of each individual participant's face recognition ability is required. The CFMT format has become widely accepted as the best available test for this purpose (e.g., ^13,14^). It offers many advantages. First, theoretically, it provides a valid test of *face* memory, rather than merely memory for a particular photograph, or an individual's general memory ability: each face is learned in three views to encourage face not picture learning; faces exclude hair; faces must later be recognised in new images (new viewpoints and lighting); and scores correlate only modestly with nonface visual memory (abstract art^15^*,* cars^16^) and barely at all with verbal memory^15,17^. Second, it has good psychometrics: the test format produces a wide range of scores in the normal population^5,18^; it has few problems with ceiling or floor effects; and it has very high internal reliability (.86-.90, various groups^15^).

Also of importance methodologically for the interpretation of the zero line in Figure 5 is that the different CFMT-tests are of equal intrinsic difficulty. This is important because mean accuracy for a race-of-face, even in own-race observers, varies with attributes of the particular face stimuli the researcher happens to have selected (e.g., how much each would "stand out in a crowd" as distinctive relative to the average appearance of people with that race/ethnicity ^19,20^; and level of physical similarity within the learning set). The CFMT variants we used have previously been shown to produce matched performance in *own*-race/ethnicity observers ^2,4,11^: for example, mean Asian-participant performance on the CFMT-Chinese equals mean Australian-Caucasian-participant performance on the CFMT-Australian. This ensures an ORE/OEE difference score (own *minus* other) of zero in Figure 5 can be taken to mean no ORE/OEE remains.

***Test order for CFMT tasks:***

For the OEE, participants completed the CFMT-Australian in one session followed by the CFMT-original in a separate session 24 hrs later. For the ORE, participants completed CFMT-Australian and CFMT-Chinese within a single session in counterbalanced order (although note order does not affect performance on CFMT format tasks^4,11^).

**Appendix 3: Classmates, Friend, and Neighbours Contact Questionnaire: Race version**

As used for participants tested on the ORE. *Note: Participants tested on Version 1* *of the contact questionnaire in years 2012, 2013 and 2015 were asked all questions below, i.e., including Sections A (Primary school contact), B (Secondary School contact), and C (Adult contact). Participants tested* *Version 2 of the contact questionnaire in year 2013 included only Section A (i.e., did not ask about Secondary or Adult contact). (also see Appendix 7)*

***A. Contact with classmates, friends, neighbours in primary school***

Now think about when you were at PRIMARY SCHOOL. Estimate what percentage (from 0 to 100%) of classmates, excluding yourself, were each of the following races:

|  | **Primary school** (approx ages 5 yrs - 12 yrs) |
| --- | --- |
| Caucasian |  |
| Asian (East or South East Asian) |  |
| Other |  |

Now think about your friends. When you were in primary school, estimate what percentage (from 0 to 100%) of your friends were each of the following races:

|  | **Primary school** |
| --- | --- |
| Caucasian |  |
| Asian (East or South East Asian) |  |
| Other |  |

Now think about the neighbourhood where you lived in primary school. Estimate what percentage (0 to 100%) of people in your neighbourhood were each of the following races:

|  | **Primary school** |
| --- | --- |
| Caucasian |  |
| Asian (East or South East Asian) |  |
| Other |  |

***B. Contact with classmates, friends, neighbours in secondary school. (Not apply to Version 2 of the contact questionnaire)***

Now think about when you were at HIGH SCHOOL. Estimate what percentage (from 0 to 100%) of classmates, excluding yourself, were each of the following races:

|  | **High school** (approx ages 13 yrs - 18 yrs) |
| --- | --- |
| Caucasian |  |
| Asian (East or South East Asian) |  |
| Other |  |

Now think about your friends. When you were in high school, estimate what percentage (from 0 to 100%) of your friends were each of the following races:

|  | **High school** |
| --- | --- |
| Caucasian |  |
| Asian (East or South East Asian) |  |
| Other |  |

Now think about the neighbourhood where you lived in high school. Estimate what percentage (0 to 100%) of people in your neighbourhood were each of the following races:

|  | **High school** |
| --- | --- |
| Caucasian |  |
| Asian (East or South East Asian) |  |
| Other |  |

**Optional variant for students who attended separate college for last two years of secondary school education:**

Some participants came from Australian states in which secondary students stay at the same school for all 6 years (referred to as "high school" above). However, in the Australian Capital Territory (where ANU is located), secondary school is split, with the first 4 years at "high school" followed by the last 2 years at "secondary college". For these students, we calculated a weight-averaged contact score, as follows:

Secondary score for ACT participant = $\frac{(High School score\times4 + College score\times2)}{6}$

***C. Contact with classmates, friends, neighbours as an adult***

Now think about yourself NOW (as an adult). Estimate what percentage (from 0 to 100%) of the university classmates you see, excluding yourself, were each of the following races:

|  | **NOW - university classmates** |
| --- | --- |
| Caucasian |  |
| Asian (East or South East Asian) |  |
| Other |  |

Now think about your friends. Estimate what percentage (from 0 to 100%) of your friends NOW are each of the following races:

|  | **NOW - friends** |
| --- | --- |
| Caucasian |  |
| Asian (East or South East Asian) |  |
| Other |  |

Now think about the neighbourhood where you live NOW. Estimate what percentage (0 to 100%) of people in your neighbourhood are each of the following races:

|  | **NOW - neighbourhood** |
| --- | --- |
| Caucasian |  |
| Asian (East or South East Asian) |  |
| Other |  |

**Appendix 4: Classmates, Friend, Neighbours Contact Questionnaire: Ethnicity version**

As used for participants tested on the OEE.

***Within-Caucasian ethnicity contact questions***

We are now going to ask you some questions about experience with various types of other Caucasians, which will require you to think about these people's appearance or background in terms of which parts of UK/Europe/Middle East their ancestry was from. For these questions, if we ask you about a "percentage" we mean percentage of the *Caucasians* you saw/knew (i.e., *not* a percentage of *all* the people you saw/knew).

1. On a scale of 0-100% what percentage of the *Caucasian* students were of a UK/ Irish/ Northern European ancestral background? This would include English, Irish, Scottish, Welsh, Swedish, German etc.
   1. when in primary school___________%
   2. high school___________%
   3. college___________%
2. On a scale of 0-100% what percentage of Caucasian students were of a Southern European or Middle Eastern background (e.g. Italian/ Greek/ Spanish/ South American/ Israeli/ Lebanese etc.)?
   1. when in primary school___________%
   2. high school___________%
   3. college___________%
3. If you are currently studying, provide an estimate of the percentage of Caucasian students in your classes who are
   1. of UK/Irish/northern European ancestry _________%
   2. of Southern European/ Middle Eastern ancestry _________%
4. On a scale of 0-100%, what percentage of your Caucasian *friends* were of a Southern European/ Middle Eastern ancestry?
   1. when in primary school___________%
   2. high school___________%
   3. college___________%
   4. now___________%
5. On a scale of 0-100%, what percentage of your Caucasian *friends* were of a UK/Irish/northern European ancestry?
   1. when in primary school___________%
   2. high school___________%
   3. college___________%
   4. now___________%
6. When answering the questions in this section;
   1. Did you feel capable of making the distinction about ancestry that we asked you to (i.e., UK/Irish/Northern European versus Southern European/ Middle Eastern)? Yes/ No
   2. In general, do you feel that you can decide, reasonably accurately, a person's ancestry in those terms (i.e., UK/Irish/Northern European versus Southern European/ Middle Eastern) based on their facial appearance? Yes/ No

**Appendix 5: Adapted 'Hancock & Rhodes' (H&R) Childhood and Adult Contact Questionnaires**

Notes: 1. The questionnaires we used (as below) were adaptions of the questionnaire originally developed by Hancock and Rhodes^1^. In the original version, the age at which the contact was obtained is not specified, and participants can potentially respond based on their current, past, or total lifetime experience. We developed the childhood-contact and adult-contact variants^2^.

2. The text appearing in red and/or bold below appeared in that format and colour when seen by participants.

***A. H&R Childhood contact questionnaire:***

For the following questionnaire, we would like you to indicate how well the following statements represent the type of interactions you had with Asian and Caucasian people **in your childhood (i.e. 0-12 years old).**

Please indicate the extent to which each statement represents your interactions by circling the number which best represents your opinion. Note: "Asian" means East or South-East Asian (e.g., Chinese, Malay, Vietnamese etc.).

**1 = Very strongly disagree; 2 = Strongly disagree; 3 = Disagree; 4 = Agree; 5 = Strongly agree; 6 = Very strongly agree**

1. I knew lots of Asian people in my childhood.

**1 2 3 4 5 6**

1. I interacted with Caucasian people during recreational periods when I was a child.

**1 2 3 4 5 6**

1. I lived in an area where I interacted with Caucasian people when I was a child.

**1 2 3 4 5 6**

1. I lived in an area where I interacted with Asian people when I was a child.

**1 2 3 4 5 6**

1. I interacted with Asian people during recreational periods when I was a child.

**1 2 3 4 5 6**

1. I interacted with Caucasian people on a daily basis when I was a child.

**1 2 3 4 5 6**

1. I socialized a lot with Caucasian people during my childhood.

**1 2 3 4 5 6**

1. I went to a childcare/primary school where I interacted with Asian kids/students.

**1 2 3 4 5 6**

1. I socialized a lot with Asian people during my childhood.

**1 2 3 4 5 6**

1. I knew lots of Caucasian people in my childhood.

**1 2 3 4 5 6**

1. When I was a child, I generally only interacted with Asian people.

**1 2 3 4 5 6**

1. I interacted with Asian people on a daily basis when I was a child.

**1 2 3 4 5 6**

1. I went to a childcare/primary school where I interacted with Caucasian kids/students.

**1 2 3 4 5 6**

1. When I was a child, I generally only interacted with Caucasian people.

**1 2 3 4 5 6**

1. I lived in a country where the predominant race is Asian when I was a child.

**1 2 3 4 5 6**

1. I lived in a country where the predominant race is Caucasian when I was a child.

**1 2 3 4 5 6**

***B. H&R Adult contact questionnaire:***

For the following questionnaire, we would like you to indicate how well the following statements represent the type of interactions you have with Asian and Caucasian people **now (i.e., at your current age).**

Please indicate the extent to which each statement represents your interactions by circling the number which best represents your opinion. Note: "Asian" means East or South-East Asian (e.g., Chinese, Malay, Vietnamese etc.).

**1 = Very strongly disagree; 2 = Strongly disagree; 3 = Disagree; 4 = Agree; 5 = Strongly agree; 6 = Very strongly agree**

1. I know lots of Asian people.

**1 2 3 4 5 6**

1. I interact with Caucasian people during recreational periods.

**1 2 3 4 5 6**

1. I live in an area where I interact with Caucasian people.

**1 2 3 4 5 6**

1. I live in an area where I interact with Asian people.

**1 2 3 4 5 6**

1. I interact with Asian people during recreational periods.

**1 2 3 4 5 6**

1. I interact with Caucasian people on a daily basis.

**1 2 3 4 5 6**

1. I socialize a lot with Caucasian people.

**1 2 3 4 5 6**

1. I interact with Asian students at university.

**1 2 3 4 5 6**

1. I socialize a lot with Asian people.

**1 2 3 4 5 6**

1. I know lots of Caucasian people.

**1 2 3 4 5 6**

1. I generally only interact with Asian people.

**1 2 3 4 5 6**

1. I interact with Asian people on a daily basis.

**1 2 3 4 5 6**

1. I interact with Caucasian students at university.

**1 2 3 4 5 6**

1. I generally only interact with Caucasian people.

**1 2 3 4 5 6**

**Appendix 6: The marriage attitude questionnaire item**

**(willingness to marry an other-race individual)**

In our particular population of participants, we expected that few if any would endorse explicit racism (e.g., statements of the form of "race X is inferior / dirty / lazy" etc.). Thus, the marriage attitude questionnaire item was designed to separate individuals based on relatively small differences in social attitudes to other-race people. Also note that our wording "Imagine looking for a serious long-term romantic partner (e.g., to live with or marry)" was designed to be suitable for the expectations of our sample: young adult Australian university students would rarely be thinking of getting married in the near future (hence "imagine"); plus, in Australia it is common to live together either before, or instead of, getting formally married; and, the wording allows for same-sex relationships (for which marriage was not legal in Australia at time of testing) as well as opposite-sex relationships.

***For Caucasian participants:***

"Imagine looking for a serious long-term romantic partner (e.g., to live with or marry). Which statement below would best summarise your preferences about that person's race (i.e., Asian, Caucasian, Melanesian, African, etc.):

1. I would consider only someone of my own race (i.e., another Caucasian).
2. I would prefer someone of my own race, but would be willing to consider someone from another race.
3. I would consider people from any race more-or-less equally, although with a small preference for my own race.
4. I would consider people from any race equally as long as they were all equally nice, although probably it would be a bit easier if they were my own race.
5. I would be completely neutral as to the person's race.
6. I would consider people from any race equally as long as they were all equally nice, although probably it would be a bit easier if they were another race.
7. I would consider people from any race more-or-less equally, although with a small preference for another race.
8. I would prefer someone of another race, but would be willing to consider someone from my own race.
9. I would consider only someone of another race (i.e., someone who is not Caucasian)."

***For Asian participants:***

"Imagine looking for a serious long-term romantic partner (e.g., to live with or marry). Which statement below would best summarise your preferences about that person's race (i.e., Asian, Caucasian, Melanesian, African, etc.):

1. I would consider only someone of my own race (i.e., another Asian).
2. I would prefer someone of my own race, but would be willing to consider someone from another race.
3. I would consider people from any race more-or-less equally, although with a small preference for my own race.
4. I would consider people from any race equally as long as they were all equally nice, although probably it would be a bit easier if they were my own race.
5. I would be completely neutral as to the person's race.
6. I would consider people from any race equally as long as they were all equally nice, although probably it would be a bit easier if they were another race.
7. I would consider people from any race more-or-less equally, although with a small preference for another race.
8. I would prefer someone of another race, but would be willing to consider someone from my own race.
9. I would consider only someone of another race (i.e., someone who is not Asian)."

**Appendix 7: Explanation of precise sample sizes and missing data**

For the *other-ethnicity effect (OEE)* contact correlations in Figure 4, the full sample size of n=46 was reduced by missing data for Adult contact with Classmates to n=35 (see Fig 4c), because 11 participants were working rather than attending university (hence no classmates score).

For the *other-race effect (ORE)* contact correlations in Figure 4, variation in precise sample size (beyond that explained due to range issues in Table S1) are detailed in Table S18 below.

***Table S18.*** Explanation of precise sample sizes for other-race contact in Figures 4 and S2, deriving from the fact that not all contact questions were asked in all years of testing, and also that in one year the included questions varied in across lab-group versus individual-participant testing.

|  |  |  | **Number of observers asked this question (in parentheses) and whose data was analysed (in bold) for this question** | | |
| --- | --- | --- | --- | --- | --- |
| **Contact measurement** | **Qnaire versions that included this question**^[1]^ | **Where are the data used?** | **Western-raised Asians** | **Eastern-raised Asians** | **Caucasians** |
| Primary C/F/N | 1, 2 | Fig 4a, S2A | (59) **59** | (105) **0** ^[2]^ | (163) **57** ^[3]^ |
| Secondary C/F/N | 1 | Fig 4b, S2B | (57) **57** | (92) **0** ^[2]^ | (120) **120** |
| Adult C/F/N | 1 | Fig 4c, S2C | (57) **57** | (92) **92** | (120) **120** |
| Child and Adult H&R | 1, 2 | Fig 4a&c, S2A&C | (59) **59** | (92) **92** | (163) **163** |
| Time-in-West | 1, 2 | Fig 4c, S2C | N/A | (105) **90** ^[4]^ | N/A |

Notes:

[1] Version 1 of the contact questionnaire (Qnaire) included all contact measures; this complete version was used for all participants tested in 2012 and 2015, and for 113 individually-tested participants in 2013.Version 2 of the questionnaire included Primary C/F/N, and H&R, and Time-in-West, but did not ask about Secondary C/F/N or Adult C/F/N; this version was used for the remaining 59 participants tested in 2013 (specifically, all 51 participants tested in lab-groups that year, plus 8 individually-tested participants).

[2] These participants were not analysed (and thus do not appear in Figures 4 and S2) due to lack of sufficient range in contact in any of the 3 years of testing (see Table S1).

[3] Of the full sample of n=163 Caucasians with Primary contact scores, only the n=57 tested in 2015 were analysed (and thus n=57 in Figs 4 and S2) due to lack of sufficient range in contact in 2012 and 2013 samples (see Table S1).

[4] The complete sample of n=105 Eastern-raised Asians were asked the relevant question (namely to report the date they arrived in Australia) but 15 participants had missing data (i.e., did not complete this question); thus there were n=90 for whom we could compute Time-in-West.

**Appendix 8: Australian demographics and immigration history that allowed dissociation between child and adult contact**

To achieve dissociation between childhood and adult contact requires a sample containing many participants for whom the balance of face types they are exposed to has changed between childhood and adulthood. We were able to obtain suitable samples due to specific aspects of demographics in Australia, arising from a combination of: Australia's migration history; differences between city and country regions; selective government schools; and university intakes.

Concerning migration history, Australia is a previous British colony. Prior to 1950, Australia's population was almost entirely British heritage (noting the original Indigenous population was relatively small and substantially reduced following European settlement, see <http://www.abs.gov.au/ausstats/abs@.nsf/0/68AE74ED632E17A6CA2573D200110075?opendocument>; and also that Australia had no African slave trade). In the 1950s-1970s, substantial migration from Southern Europe (Southern Italy and Greece) occurred. Between 1901 and 1973 Australian had a White Australia policy, restricting migration from Asia; after the removal of this policy, migration from Asia gradually increased, to the point where people born in East Asia comprised 8.4% of the Australian population at time of testing (Australian Bureau of Statistics, 2011). Concerning differences between city and country, the majority of non-British migration (i.e., from Southern Europe and Asia) has been to the largest cities (particularly Sydney and Melbourne), with less to Canberra and Perth (our testing locations), and very little to country towns outside the cities. Concerning selective schools, Sydney has several free government schools with entry based on high academic performance, which in the last decade have had an extremely high proportion of students of East Asian origin (approximately 80% of enrolments; generally the children of first-generation immigrants). Concerning university intakes, both The Australian National University in Canberra and The University of Western Australia in Perth have a mix of city-raised and country-raised students. The ANU also has a significant intake of non-Canberra students from Sydney and Melbourne. Both universities also have a substantial number of international students from Asia, such that the proportion of Asians in the university enrolment cohort is much higher than in the general Australian population (with ANU having approximately 31% and UWA 23% total; proportion also varies somewhat across discipline-of-enrolment, being highest in disciplines least reliant on English-language ability such as accounting or mathematics, and lower in writing-heavy disciplines).

Together, these factors combine to mean that for our Australian-born samples — the Caucasian sample on the ORE, the Western-raised Asian sample on the ORE and the Northern-European (specifically British-heritage) sample on the OEE — many different patterns of lifetime exposure were possible. For example, some participants might have been raised in a country town (with no Asian exposure as a child) followed by moving to the city for university and studying accounting (high Asian exposure as an adult, due to the large number of international students in their university classes), other participants might have been raised in Canberra (with low but nonzero Asian exposure from the general population as a child) followed by studying accounting at university (high Asian exposure as an adult), some participants might have been raised in Sydney (modest Asian exposure as a child) followed by studying a language-heavy discipline such as law at university (fewer international students and thus modest Asian exposure as an adult), and some participants could have had higher Asian exposure as a child than as an adult due to attending a selective Sydney school, or being raised in an Asian family environment or in a particular Sydney suburb that was preferentially settled by Asian immigrants.

For our Asian-born sample (i.e., the Eastern-raised Asians), note that there was no similar demographic variation in childhood exposure: this group generally had no or almost no Caucasian exposure as a child. However, this was followed by normally-distributed adult Caucasian contact at university. This means that effects of adult exposure in this sample were analysable independent of childhood contact.

**REFERENCES FOR SUPPLEMENTARY INFORMATION**

1 Hancock, K. J. & Rhodes, G. Contact, configural coding and the other-race effect in face recognition. *Brit. J. Psychol.* **99**, 45-56, doi:10.1348/000712607X199981 (2008).

2 Wan, L., Crookes, K., Reynolds, K. J., Irons, J. L. & McKone, E. A cultural setting where the other-race effect on face recognition has no social–motivational component and derives entirely from lifetime perceptual experience. *Cognition* **144**, 91-115, doi:10.1016/j.cognition.2015.07.011 (2015).

3 Nunnally, J. C., Bernstein, I. H. & Berge, J. M. T. *Psychometric Theory*. Vol. 226 (McGraw-Hill, 1967).

4 McKone, E. *et al.* Face ethnicity and measurement reliability affect face recognition performance in developmental prosopagnosia: Evidence from the Cambridge Face Memory Test - Australian. *Cog. Neuropsychol.* **28**, 109-146, doi:10.1080/02643294.2011.616880 (2011).

5 DeGutis, J., Wilmer, J., Mercado, R. J. & Cohan, S. Using regression to measure holistic face processing reveals a strong link with face recognition ability. *Cognition* **126**, 87-100, doi:10.1016/j.cognition.2012.09.004 (2013).

6 Ley, P. *Quantitative aspects of psychological assessment*. (Duckworth, 1972).

7 Brigham, J. C. College students’ racial attitudes. *J. of Appl. Soc. Psychol.* **23**, 1933-1967, doi:10.1111/j.1559-1816.1993.tb01074.x (1993).

8 Brigham, J. C., Bennett, L. B., Meissner, C. A. & Mitchell, T. L. in *The Selected Works of Christian A. Meissner, Ph.D.* 28 (2007).

9 Slone, A. E., Brigham, J. C. & Meissner, C. A. Social and cognitive factors affecting the own-race bias in Whites. *Basic & Appl. Soc. Psychol.* **22**, 71-84, doi:10.1207/S15324834BASP2202_1 (2000).

10 Wan, L. *et al.* Face-blind for other-race faces: Individual differences in other-race recognition impairments. *J. Exp. Psychol.: Gen.* **146**, 102-122, doi:10.1037/xge0000249 (2017).

11 McKone, E. *et al.* A robust method of measuring other-race and other-ethnicity effects: The Cambridge Face Memory Test format. *PLoS One* **7**, e47956, doi:10.1371/journal.pone.0047956 (2012).

12 Duchaine, B. C. & Nakayama, K. The Cambridge face memory test: Results for neurologically intact individuals and an investigation of its validity using inverted face stimuli and prosopagnosic participants. *Neuropsychologia* **44**, 576-585, doi:10.1016/j.neuropsychologia.2005.07.001 (2006).

13 Rhodes, G., Jeffery, L., Taylor, L., Hayward, W. G. & Ewing, L. Individual differences in adaptive coding of face identity are linked to individual differences in face recognition ability. *J. Exp. Psychol.: Hum. Percept. & Perform.* **40**, 897-903, doi:10.1037/a0035939 (2014).

14 DeGutis, J., Mercado, R. J., Wilmer, J. & Rosenblatt, A. Individual differences in holistic processin predict the own-race advantage in recognition memory. *PLoS One* **8**, e58253, doi:10.1371/journal.pone.0058253 (2013).

15 Wilmer, J. B. *et al.* Human face recognition ability is specific and highly heritable. *Proc. Nat. Acad. of Sci.* **107**, 5238-5241, doi:10.1073/pnas.0913053107 (2010).

16 Dennett, H. W. *et al.* The Cambridge Car Memory Test: A task matched in format to the Cambridge Face Memory Test, with norms, reliability, sex differences, dissociations from face memory, and expertise effects. *Beh. Res. Methods* **44**, 587-605, doi:10.3758/s13428-011-0160-2 (2012).

17 Bowles, D. C. *et al.* Diagnosing prosopagnosia: Effects of ageing, sex, and participant-stimulus ethnic match on the Cambridge Face Memory Test and Cambridge Face Perception Test. *Cog. Neuropsychol.* **25**, 423-455, doi:10.1080/02643290903343149 (2009).

18 Palermo, R. *et al.* Do people have insight into their face recognition abilities? *Quarterly J. Exp. Psychol.* **70**, 218-233, doi:10.1080/17470218.2016.1161058 (2016).

19 Gilchrist, A. & McKone, E. Early maturity of face processing in children: Local and relational distinctiveness effects in 7-year-olds. *Vis. Cog.* **10**, 769-793, doi:10.1080/13506280344000022 (2003).

20 Valentine, T. & Bruce, V. The effects of distinctiveness in recognising and classifying faces. *Perception* **15**, 525-535, doi:10.1068/p150525 (1986).
